# An Experimentally Defined Hypoxia Gene Signature in Glioblastoma and Its Modulation by Metformin

**DOI:** 10.3390/biology9090264

**Published:** 2020-09-02

**Authors:** Marta Calvo Tardón, Eliana Marinari, Denis Migliorini, Viviane Bes, Stoyan Tankov, Emily Charrier, Thomas A McKee, Valérie Dutoit, Pierre-Yves Dietrich, Erika Cosset, Paul R Walker

**Affiliations:** 1 Center for Translational Research in Onco-Hematology, Division of Oncology, Geneva University Hospitals and University of Geneva, 1211 Geneva, Switzerland; mcalvotardon@gmail.com (M.C.T.); eliana.marinari@unige.ch (E.M.); viviane.bes@unige.ch (V.B.); stoyan.tankov@unige.ch (S.T.); emily.charrier@unige.ch (E.C.); valerie.dutoit@unige.ch (V.D.); Pierre-Yves.Dietrich@hcuge.ch (P.-Y.D.); erika.cosset@unige.ch (E.C.); 2 Department of Oncology, Clinical Research Unit, Dubois Ferrière Dinu Lipatti Research Foundation, Geneva University Hospitals, 1205 Geneva, Switzerland; denis.migliorini@unige.ch; 3 Division of Clinical Pathology, Geneva University Hospitals, 1211 Geneva, Switzerland; thomas.a.mckee@hcuge.ch

**Keywords:** hypoxia, physioxia, glioblastoma, glioblastoma microenvironment, metformin, hypoxia gene signature

## Abstract

Glioblastoma multiforme (GBM) is the most common and aggressive primary brain tumor, characterized by a high degree of intertumoral heterogeneity. However, a common feature of the GBM microenvironment is hypoxia, which can promote radio- and chemotherapy resistance, immunosuppression, angiogenesis, and stemness. We experimentally defined common GBM adaptations to physiologically relevant oxygen gradients, and we assessed their modulation by the metabolic drug metformin. We directly exposed human GBM cell lines to hypoxia (1% O_2_) and to physioxia (5% O_2_). We then performed transcriptional profiling and compared our in vitro findings to predicted hypoxic areas in vivo using in silico analyses. We observed a heterogenous hypoxia response, but also a common gene signature that was induced by a physiologically relevant change in oxygenation from 5% O_2_ to 1% O_2_. In silico analyses showed that this hypoxia signature was highly correlated with a perinecrotic localization in GBM tumors, expression of certain glycolytic and immune-related genes, and poor prognosis of GBM patients. Metformin treatment of GBM cell lines under hypoxia and physioxia reduced viable cell number, oxygen consumption rate, and partially reversed the hypoxia gene signature, supporting further exploration of targeting tumor metabolism as a treatment component for hypoxic GBM.

## 1. Introduction

Glioblastoma (GBM) is a diffuse astrocytic tumor, which in the 2016 WHO classification is divided into those that are isocitrate dehydrogenase (*IDH*)-wildtype and those that are *IDH*-mutant [1]. It is the most common and aggressive primary tumor in the central nervous system [2] and median survival of GBM patients is only 12–15 months [3], despite a standard of care consisting of surgical resection and radio-chemotherapy. An important characteristic of these tumors is the high level of heterogeneity, both intertumoral [4,5] and intratumoral [6,7].

A common feature found in most solid tumors is the presence of hypoxia as a result of rapid cancer cell proliferation and aberrant vasculature that is unable to maintain oxygen supply [8]. Tumor hypoxia drives malignancy by promoting chemo- and radiotherapy resistance, an immunosuppressive microenvironment, cancer cell stemness, angiogenesis, and metabolic modulation [9,10,11]. The study of tumor hypoxia in vitro frequently uses cell cultures exposed to atmospheric conditions (21% O_2_) as a control, although this does not represent any physiological oxygen fraction found in vivo [12] and does not always recapitulate cellular functions under physioxia [13]. Physiological oxygen availability is tissue-dependent, with 2%–9% O_2_ (10–40 mmHg) being reported for the healthy brain [14]. Oxygen fractions used to refer to tumor hypoxia vary between studies, but 0.5%–2% O_2_ (i.e., less than physiological values, and thereby inadequate oxygenation) are observed in vivo in the tumor bed and are used experimentally in vitro [15,16]. One of the key regulators of the hypoxia response is hypoxia-inducible factor (HIF)-1α [17], but HIF-independent cellular pathways have also been reported [18,19].

Aberrant signaling pathways such as mTOR or pro-tumoral functions such as VEGF release have been individually targeted in GBM therapy, using rapamycin (or its derivatives) or bevacizumab, respectively, but have shown a limited influence on overall survival of GBM patients to date [20,21]. Metformin, a type 2 diabetes drug, has been shown to decrease the risk of developing certain types of cancer [22], and can potentially target mTOR signaling and also reprogram oxygen metabolism, thereby reducing hypoxia in the tumor microenvironment. Metformin has been shown to improve the anti-tumor immune response in several mouse tumor models [23,24,25]. In the context of GBM, metformin can inhibit cell growth through mTOR inhibition, and has been observed to enhance the therapeutic effect of temozolomide in human xenografts [26]. The effects of metformin have therefore generated interest in the anti-cancer effects of metabolic drugs, but questions remain concerning their impact on GBM cells, particularly under in vivo-relevant oxygen deprivation.

In the treatment of GBM, a better understanding of its genetic, epigenetic, and/or transcriptional characteristics could help to identify markers or signatures that predict outcomes or responses to specific therapies, as exemplified by MGMT promoter methylation status, which predicts responses to temozolomide [27]. More recently, gene mutations and expression profiles are being studied to associate specific gene signatures with clinical outcomes [28,29], including hypoxia-induced gene signatures in multiple cancer types [8,30]. Here, we evaluated the GBM response to low levels of oxygen and, despite GBM heterogeneity, we identified a common hypoxia gene signature that was determined experimentally and that was associated with pseudopalisading and necrotic areas of GBM from patient data. The signature correlated with expression of certain glycolysis- and immune-related genes, and importantly, survival. We validated the use of metformin to force metabolic changes in GBM cells and to reduce oxygen consumption and numbers of viable tumor cells.

## 2. Materials and Methods

### 2.1. In Vitro Cultures

Human Ge904 at passage (p11), Ge835 (p8), and Ge898 (p10) were obtained in house from resection of primary *IDH* wildtype (WT) GBM. Research use of this human material was approved by the local Institutional Review Board and Ethics committee, with signed informed consent obtained for all patients. LN18 (p560) and LN229 (p209) were obtained from American Type Culture Collection (ATCC), U87 (unknown passage number), and U251 (p590) were obtained from European Collection of Authenticated Cell Cultures (ECACC); mouse SB28 was kindly provided by H. Okada, University of California, San Francisco (UCSF), USA [31]; and GL261-OVA was kindly provided by O. Grauer, University Hospital of Münster, UKM, Germany [32]. Normal human astrocytes were obtained from ScienCell. All cell lines were cultured in serum-containing Dulbecco’s Modified Eagle Medium (DMEM)-based media and passaged every 2–3 days. GBM cell lines were exposed to atmospheric O_2_ conditions in a conventional hood and incubator, or to 1% O_2_ or 5% O_2_ using the Ruskinn 300 InVivO2 hypoxia workstation (Baker) for 48 h. Media were pre-equilibrated to the desired oxygen level by flushing with the corresponding gas mix. All cell lines tested negative for mycoplasma.

All subjects gave their informed consent for inclusion before they participated in the study. The study was conducted in accordance with the Declaration of Helsinki, and the protocol was approved by the Ethics Committees of Geneva University Hospitals and the Canton of Geneva (CCER) (03-126).

### 2.2. Sequencing and Polymerase Chain Reaction (PCR)

Total RNA was extracted using Qiagen RNeasy Kit, following manufacturer′s instructions. Gene expression by microarray was employed for Ge835, Ge898, Ge904, LN18, and LN229 using Microarray PrimeView Human Gene Expression Array (Affymetrix) probes and associated analysis files, with gene annotation for each set of probes (PrimeView Human Gene Expression Array Library files, version 2014).

qPCR of the hypoxia signature genes was performed to quantify mRNA levels of metformin or vehicle-treated cells exposed to hypoxia or physioxia. Briefly, DNase-treated RNA was used to synthesize cDNA (PrimerScript RT; Takara Bio Inc., Shiga, Japan) The genes analyzed and the primers used are indicated in Table 1.

### 2.3. TP53 Analysis

DNA from cell lines Ge898 and Ge904 was sequenced using an Illumina NextSeq 500 instrument using standard protocols. Briefly, libraries were prepared from 100 ng of genomic DNA that was fragmented using a Kapa hyperplus kit (Roche, Basel CH, Switzerland). TP53 sequences were captured using a custom SureSelect panel (Agilent, Santa Clara, CA, USA). Libraries were prepared using an Illumina NextSeq 550/500 v2.5 sequencing reagent kit (Illumina, San Diego, CA, USA) and sequencing was performed on an Illumina NextSeq 500 instrument (Illumina, San Diego, CA, USA) with pair end reads of 150 bp. Variants were called using a custom bioinformatics pipeline based on MuTect 2 [33]. The information regarding the TP53 status of the other GBM cell lines was extracted from the literature for Ge835 [34], SB28 [35], and GL261 [36], or from available databases [37].

### 2.4. Western Blot

Fifteen μg of whole protein lysates (NP-40-based lysis buffer) or nuclear fractions (NE-PER™ Nuclear and Cytoplasmic Extraction Reagents, ThermoFisher, Waltham, MA, USA) were loaded onto 12.5% SDS-PAGE gel and transferred onto nitrocellulose membranes. Membranes blocked with 5% non-fat dry milk were incubated with the following antibodies: rabbit anti-HIF-1α (Bethyl, Montgomery, TX, USA), mouse anti-TBP (Novus Biologicals, Littleton, CO, USA), followed by goat anti-rabbit IgG-HRP (Sigma, St. Louis, MO, USA) or goat anti-mouse IgG-HRP (Sigma). Enhanced chemiluminescent (ECL, Novus Biologicals, Littleton, CO, USA) detection (SuperSignal West Pico, ThermoFisher) was used to observe reactive bands.

### 2.5. In Vitro Assays

All assays were performed for 48 h under the corresponding oxygenation conditions. Viable cell numbers were assessed using CellTiter Glo (Promega, Madison, WI, USA), following manufacturer’s protocols, with luminescence measured using a Cytation3 reader (BioTek, Winooski, VT, USA). For metabolic assays, oxygen consumption rate (OCR) and extracellular acidification rate (ECAR) were measured using a Seahorse XFe96 Analyzer (Agilent) installed in a hypoxia workstation to perform experiments at the indicated O_2_ concentrations. To achieve an even distribution of cells within wells, plates were rocked for 20–40 min. The plate was then incubated at 37 °C overnight to allow the cells to adhere. The following day, growth media was exchanged with XF media (DMEM-based Phenol Red-free media, Agilent) with additional 1 g/L glucose, 2 mM glutamine and 1 mM sodium pyruvate. The plate was then incubated at 37 °C and atmospheric CO_2_ for 1 h. The Cell Mito Stress kit (Agilent) was used following the manufacturer’s instructions. Briefly, 2.25 μM Oligomycin (to inhibit ATP synthase) was injected first in the assay following basal measurements in order to reduce OCR. Then 2.25 μM 7 Carbonyl cyanide-4 (trifluoromethoxy) phenylhydrazone (FCCP) was injected to disrupt the mitochondrial membrane potential and to calculate spare respiratory capacity, defined as the difference between maximal respiration and basal respiration. Finally, 1.13 μM Rotenone/Antimycin A was injected (inhibiting complex I and complex III) to shut down mitochondrial respiration and to enable the calculation of nonmitochondrial respiration driven by processes outside the mitochondria. Results from all wells were normalized to cell number.

### 2.6. Bionformatic and Statistical Analysis

The Microarray was performed with 3 Affymetrix Prime View chips, 15 samples per chip, 5 groups of samples with 3 replicates and 3 treatments. The robust multi-array average (RMA)-normalized intensities were analyzed for differential expression [38]. The following comparisons were done on each of the 3 independent experiments with a *t*-test for each cell line and on all samples with a paired sample ANOVA (Fold change (FC) ≥ 1.3 and FC ≤ −1.3, *p* < 0.05), using Partek^®^ Genomics Suite^®^ software, version 6.6. The following comparisons were done: 1 versus 5, 1 versus 21, and 5 versus 21. The hypoxia gene signature obtained from the ANOVA analysis comprised 33 coding genes with gene ontology annotation according to the PrimeView Human Gene Expression Array Library used.

In silico analysis included several datasets: for GBM, The Cancer Genome Atlas (TCGA, *n* = 528 grade IV) [39] and IvyGAP (*n* = 270 grade IV) [40] were used; for high grade glioma including GBM, Rembrandt (*n* = 267, of which *n* = 79 grade III and *n* = 188 grade IV), Phillips (*n* = 100, of which *n* = 24 grade III and *n* = 76 grade IV) [5], and Freije (*n* = 85, of which *n* = 26 grade III and *n* = 59 grade IV) [41] were used. The GlioVis data portal was employed for visualization and analysis of brain tumor expression datasets, and downloading of clinical data and normalized counts [42]. Data cleaning and merging was performed in R version 3.3.2, using library dplyr to join phenotype and expression data for TCGA, Freije, Phillips, and Rembrandt datasets [43,44]. K-means clustering allowed us to group patients in high risk or low risk categories, according to signature expression (one minus Pearson correlation metric) and the similarity matrix was used to compare the expression of different gene families in the signature and to estimate the correlation (Pearson correlation metric). Analysis and graphs were generated using Morpheus [45]. Kaplan–Meyer survival analysis and curves were done with Prism 8, Version 8.2.1, 2019.

Gene set enrichment analysis (GSEA) was performed as previously described, using GSEA v.4.0.1 graphical user interface (GUI) and the hallmarks gene set from MSigDB v7.0 [43], with the following parameters: Signal2Noise metric, weighted scoring, and *n* = 1000 permutations. TCGA patient characteristics were compared using the Chi-Square test by IBM SPSS^®^ statistics, version 25.0.5. DAVID functional annotation clustering and chart analysis was performed with version 6.8 (selected options: BBID, BIOCARTA, KEGG_PATHWAY, thresholds: count = 2, EASE = 0.1).

## 3. Results

**Figure 1:**
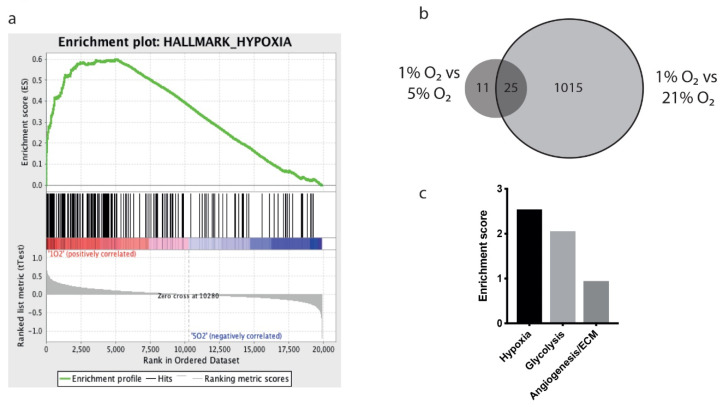
Whole transcriptome analysis of Ge835, Ge898, Ge904, LN18, and LN229 glioblastoma multiforme (GBM) cell lines cultured under hypoxia (1% O_2_), physioxia (5% O_2_), or hyperoxia (21% O_2_) for 48 h. (**a**) Gene set enrichment analysis for hypoxia geneset comparing transcriptional profiles of hypoxia versus physioxia; (**b**) Venn diagram of comparisons between hypoxia and physioxia, and hypoxia and hyperoxia; (**c**) Enrichment scores of gene families from DAVID analysis.

We exposed five human GBM lines (Ge835, Ge898, Ge904, LN18, and LN229) to various oxygen conditions: inadequate oxygenation (hypoxia, 1% O_2_), physiological (physioxia, 5% O_2_), and atmospheric (hyperoxia, 21% O_2_) conditions, and performed transcriptional profiling using the Affymetrix Microarray. Comparing 1% O_2_ to 5% O_2_ showed an enrichment in the hallmark hypoxia gene set after performing gene set enrichment analysis (GSEA) (Figure 1a). Our experimental approach consisted of directly modulating oxygenation levels, thereby reproducing in vivo attainable oxygen gradients. This allowed us to identify transcriptional changes reported in GSEA [43]. We confirmed hypoxia adaptation by quantifying nuclear stabilization of HIF-1α by Western blot analysis (Figures S1a and S4).

Comparing hypoxia (1% O_2_) to hyperoxia (21% O_2_), we identified 1040 common differentially expressed genes (ANOVA), whereas comparing hypoxia to physioxia (5% O_2_) revealed only 36 differentially expressed genes (Figure S1d). Twenty-five of these 36 genes (69%) were common to the 21% to 1% O_2_ comparison, but 11 genes were unique to the 5% to 1% O_2_ comparison (Figure 1b). This suggests that using atmospheric conditions as a control not only leads to an overestimation of the adaptation of GBM cells to hypoxia, but might also obscure important biological processes taking place under physiological conditions.

We were able to build a hypoxia gene signature by selecting the differentially expressed genes between hypoxia and physioxia from the ANOVA analysis, considering all five cell lines together (FC ≥ 1.3 and FC ≤ −1.3, *p* < 0.05, Supplementary File 1). Performing DAVID analysis [46], we determined that this experimentally defined signature was significantly enriched for hypoxia, glycolysis, and angiogenesis and extracellular matrix gene clusters (Figure 1c). Of note, 18 of the genes in the signature are not reported to have a hypoxia-responsive element (HRE) sequence [47,48], and therefore may represent HIF-independent hypoxia-regulated responses (Figure S1e). However, the GBM lines analyzed showed a significant level of heterogeneity. Indeed, unsupervised clustering of the transcriptional data grouped the samples by cell line, rather than by the effect of hypoxia (Figure S1c, Table S1). In addition, when different cell lines were separately analyzed for differential expression comparing hypoxia to physioxia, there were no common hits between the five cell lines (Figure S1b, Supplementary File 2).

**Figure 2:**
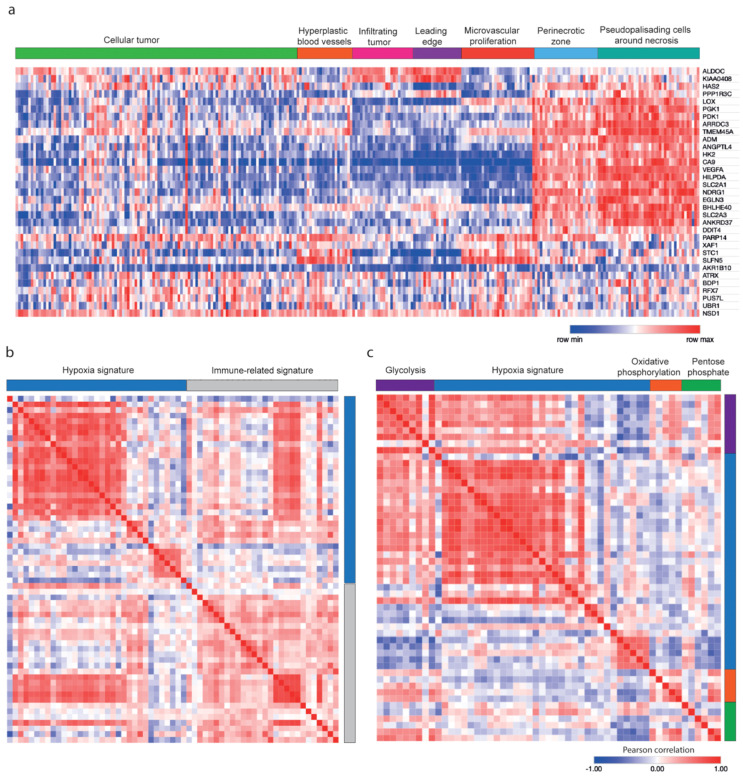
Expression and correlation of the hypoxia gene signature using the Ivy-GAP database. (**a**) Expression of the hypoxia gene signature in different areas of human GBM (*n* = 270) biopsies; (**b**,**c**) Correlation matrix of the hypoxia signature with (**b**) immune-related genes or (**c**) three metabolic pathways gene lists. List of immune-related genes (from left to right and top to bottom): *ARG2*, *ARG1*, *TGFB1*, *IDO1*, *IL10*, *CD163*, *MRC1*, *CCR4*, *CCR7*, *CD80*, *CD3G*, *CD3D*, *CD3E*, *GZMB*, *PRF1*, *CD86*, *IL1A*, *IL6*, *IL1B*, *IL8*, *CCL20*, *TNF*, *IFNG*, *IL2*, *ICAM1*, *ITGAL*, *ITGA4*, *ITGB2*.

**Figure 3:**
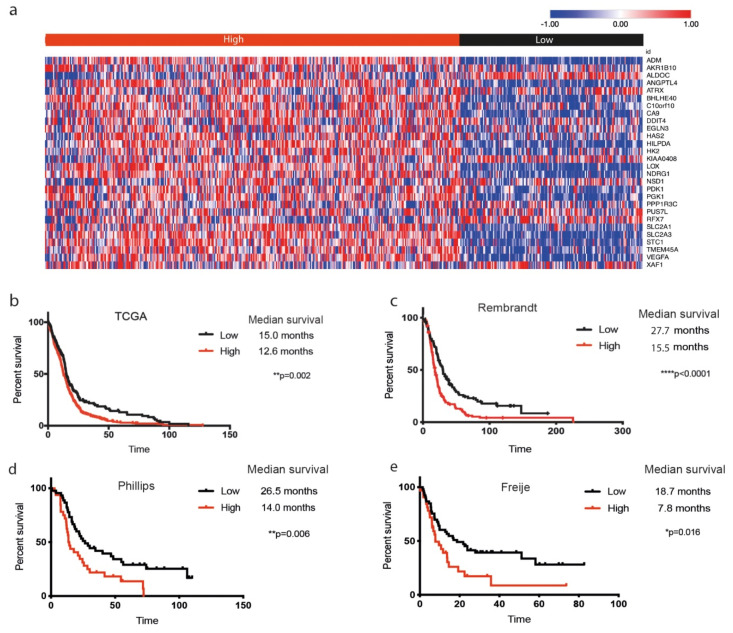
Correlation of the hypoxia signature with survival of high-grade glioma patients. (**a**) Expression of the hypoxia gene signature (z-score) across patients from TCGA database (*n* = 528), segregated by high and low expression of the signature; (**b**–**e**) Kaplan–Meier survival curves corresponding with high (red) or low (black) expression of the hypoxia signature in (**b**) TCGA (*n* = 528), (**c**) Rembrandt (*n* = 267), (**d**) Phillips (*n* = 100), and (**e**) Freije (*n* = 85) databases.

Interestingly, the signature identified with this experimental approach, directly modulating the availability of oxygen in our GBM cell lines, was highly enriched in predicted hypoxic regions from existing GBM databases. We interrogated our hypoxia signature in the Ivy-GAP platform [40,42], a GBM dataset originating from biopsies and microdissections. The hypoxia signature was highly expressed within perinecrotic and pseudopalisading areas of tumors (predicted to include hypoxic zones) confirming that our signature reflects in vivo observed features (Figure 2a). Our signature was strongly correlated with an inflammatory phenotype that included expression of genes encoding IL-1β, IL-6, and IL-8 (Figure 2b), and with the glycolytic pathway (Figure 2c). Analysis of the microarray data of GBM cell lines also showed a correlation of our hypoxia signature with expression of genes related to inflammation, suggesting that the tumor cells may have contributed to the database findings, in which gene expression of both tumor cells and infiltrating immune cells is measured (data not shown). GBM (TCGA Table 2) and high-grade glioma patients (from Rembrandt, Phillips, and Freije databases) [5,39,41,49] were clustered in two groups according to signature expression, based on a supervised clustering method that allows group partitioning such as k-means cluster analysis (k = 2, one minus Pearson correlation). Importantly, a high expression of our signature correlated with poor survival (Figure 3 and Table S4). The TCGA dataset included IDH mutational status. A high expression of our signature was strongly correlated with the absence of IDH mutations (Table 2). Moreover, our signature was highly associated with survival in a univariate analysis, but not in a multivariate analysis combined with IDH status, suggesting that it could be an important feature linked to the genetic characteristics of the tumor and not an independent prognostic biomarker (Table S3).

**Figure 4:**
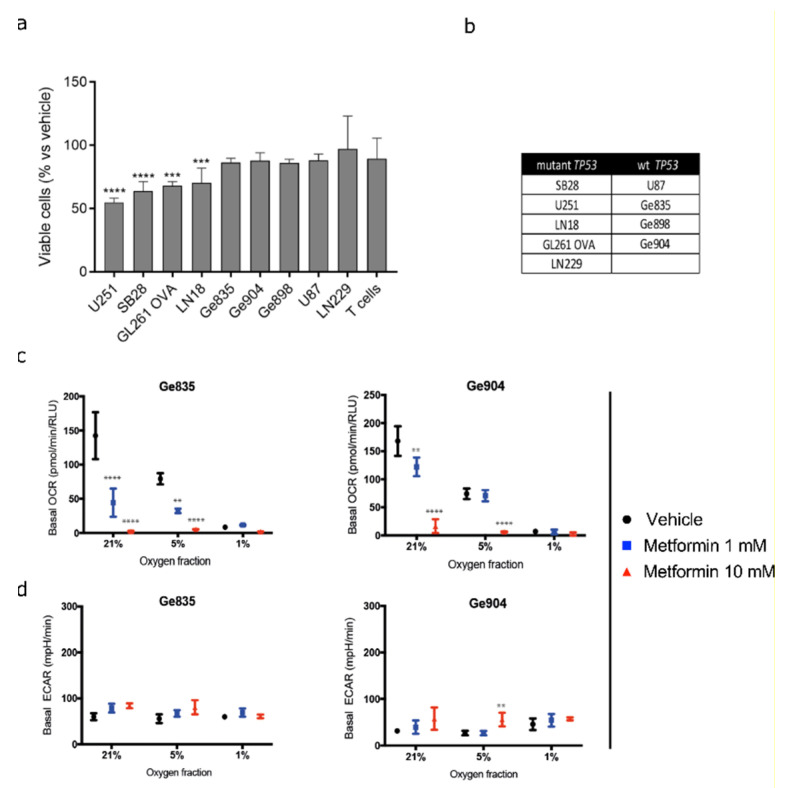
Functional assays of human and mouse GBM cell lines exposed to metformin. (**a**) Effect of 10 mM metformin on viable cell number under physioxia; (**b**) Table indicating *TP53* mutation status of all GBM cell lines used; mutant or wild type (WT); (**c**) Basal oxygen consumption rate (OCR) and (**d**) basal extracellular acidification rate (ECAR) of Ge835 and Ge904 GBM cell lines in vitro at 21%, 5%, and 1% O_2_, with the indicated concentrations of metformin (mean of 3 independent experiments +/- SD; 2-way ANOVA, Sidak’s adjusted *p*-value. ** *p* < 0.01, *** *p* < 0.001, **** *p* < 0.0001).

Tumor hypoxia could potentially be modulated by the metabolic drug metformin. We therefore investigated its effects on GBM viable cell number and oxygen consumption rate, which were previously only described using non-physiological oxygen conditions. In these in vitro assays, we used several human GBM cell lines (including Ge835, Ge904, and LN18, for which transcriptional profiling had been performed), and two mouse glioma models (SB28 and GL261-OVA). As expected, metformin reduced the number of viable cells of several of the cell lines in culture (based on ATP quantitation) under physioxia (Figure 4a), and we confirmed that this tendency was maintained under hyperoxia and hypoxia (Figure S2a). There was no significant influence of metformin on normal T cells, but there was a modest, dose-dependent reduction in the number of viable cells from cultures of non-malignant astrocytes (Figure S2b). The influence of metformin on numbers of viable human GBM line cells under physioxia was principally due to a cytotoxic mechanism, through apoptosis and necrosis (Figure S2c), rather than cytostasis, as proliferation was not reduced (data not shown).

Since mutational status can have an effect on metabolism [50], we assessed whether mutations in *PTEN*, *IDH*, and *TP53*, or the promoter methylation status of the gene encoding for the repair enzyme O6-methylguanine-DNA methyltransferase (*MGMT*), had an effect on metformin responsiveness. Although those cell lines (U251, SB28, GL261 OVA, and LN18) that had a statistically significant reduction in viable cell numbers in response to metformin when assessed by ATP content (*p* < 0.001) were all *TP53*-mutant (Figure 4b), LN229, which was also *TP53*-mutant, showed no sensitivity to metformin in this assay. However, when the more sensitive flow cytometry-based assay assessing apoptotic and necrotic cells was used, LN229, as well as Ge904 and Ge898 (both *TP53* WT), were all significantly affected by metformin treatment (Figure S2c), establishing no correlation between the response to metformin under physioxia and *TP53* mutation status. We cannot exclude the potential importance of mutated *IDH*, as none of the lines harbored *IDH* mutations; however, the response to metformin under physioxia was not associated with *MGMT* promoter methylation or *PTEN* mutation status (Fischer’s exact test, *p* > 0.05).

We evaluated oxygen consumption rate (OCR) and extracellular acidification rate (ECAR) on metformin-treated GBM cell lines under hypoxia, physioxia, and hyperoxia. We first validated the reduction in OCR induced by metformin under hyperoxic conditions (Figure 4c, Figure S3a,b). Metformin reduced OCR under physioxia in human GBM cell lines in vitro (Figure 4c, Figure S2a,b). Under hypoxic conditions, the availability of oxygen was clearly a limiting factor in these measurements, indicated by the lower OCR (Figure 4c), which did not permit us to observe significant changes after treatment. Overall, our results suggest that metformin shifted metabolism; this also resulted in a modest trend towards an increased ECAR, but only with high doses, in LN18, Ge904, U251, SB28, and GL261-OVA cell lines (Figure 4d, Figure S2c).

**Figure 5:**
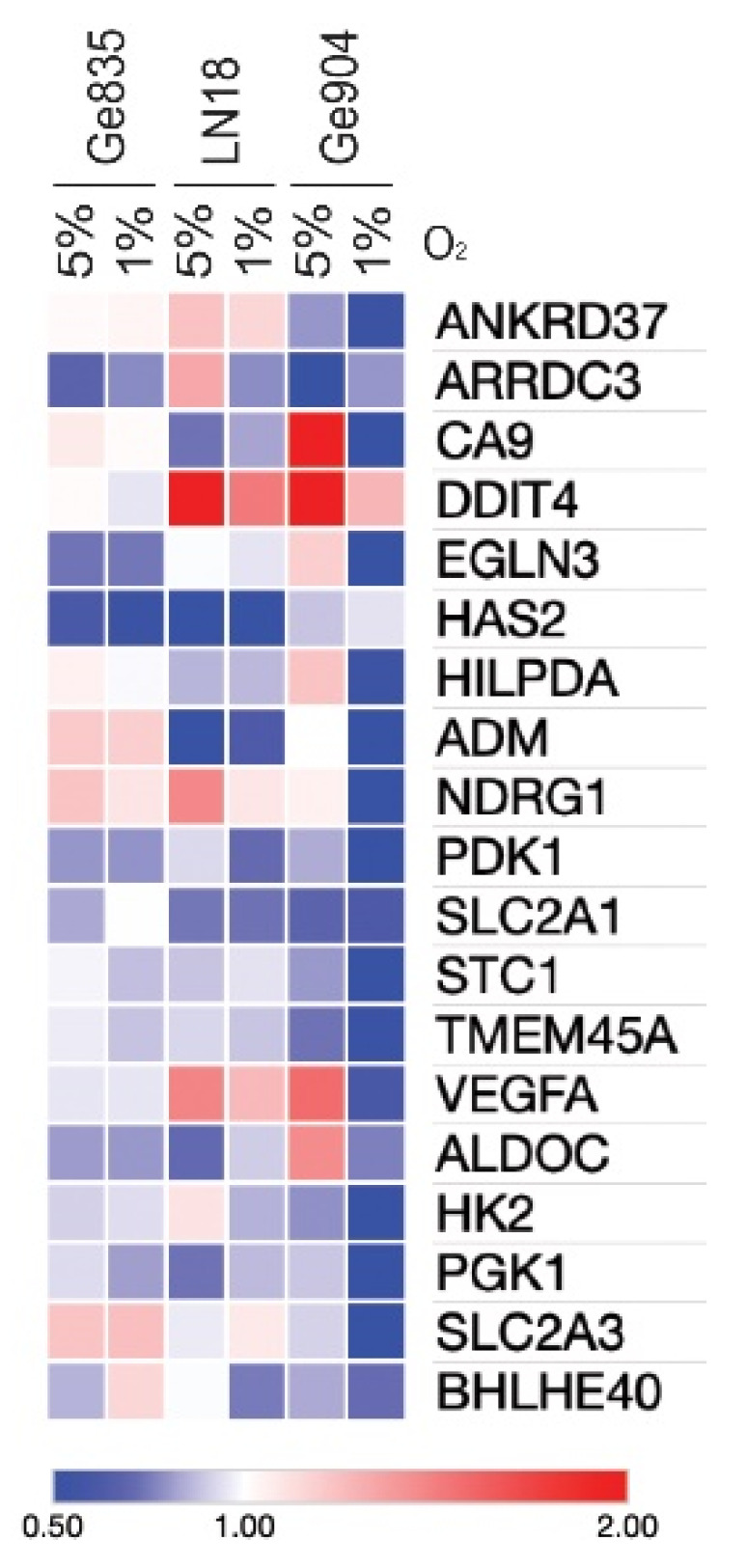
Metformin modulation of the upregulated genes of our hypoxia gene signature. Heat map represents fold-changes of expression between metformin-treated (10 mM, 48 h) versus vehicle-treated cells (red, FC > 2; blue, FC < 0.5). mRNA expression measured by qPCR of upregulated genes from the hypoxia gene signature on Ge835, LN18, and Ge904 treated with metformin or vehicle and exposed to physioxia or hypoxia. Fold change values are tabulated in Table S2.

To further assess the consequences of metformin treatment, we tested three of the human GBM lines for which we had both transcriptome data and metabolic analyses (Ge835, LN18, Ge904) and measured expression of our hypoxia signature after metformin treatment. There was a downregulation of most genes of the signature after exposure of these GBM lines to hypoxia or physioxia, although there was a certain level of heterogeneity (Figure 5, Table S2). This effect was more pronounced under hypoxia, compared to physioxia. Some genes, such as *DDIT4* and *VEGFA*, instead showed upregulation in response to metformin treatment, although this was mostly cell line-specific.

## 4. Discussion

The intertumoral heterogeneity of GBM is a known and expected feature. Here we provide detailed evidence of intertumoral heterogeneity at the transcriptional level by performing in vitro hypoxia studies using several human-derived cell lines, which allowed us to identify a robust common hypoxia signature (Figure S1d), despite the heterogeneity. Importantly, we used 5% O_2_ as a physiological oxygen control, rather than 21% O_2_, more accurately representing the in vivo oxygenation levels, which can influence viable cell number, metabolism, and mitochondrial function [13]. By analyzing differential gene expression at three different oxygen concentrations (Figure 1b), it is clear that the vast majority of transcriptional changes had already occurred under physioxia, with a very modest number of genes differentially regulated between 5% O_2_ and 1% O_2_. Surprisingly, such comparisons are rarely made in hypoxia studies, although we observed effects of a similar magnitude when studying CD8^+^ T cells [15]. It is probable that changes in gene expression could also occur under other physiologically attainable oxygen fractions, which could be modeled by culture between 5% and 21% O_2_, and which could be controlled by different isoforms of HIF that differ in their stability under oxygen in a time- and concentration-dependent manner [51]. Thus, a hypoxia response can be considered as a spectrum of adaptations, which we have sampled at certain points in our study. Nevertheless, we identified a hypoxia gene signature after ANOVA analysis considering multiple GBM cell lines, and selecting the differentially expressed genes between 5% and 1% O_2_.

The hypoxia gene signature was obtained from an experimental approach, directly evaluating the effect of hypoxia on glioma cells. Five different cell lines were used in this study and the signature was the result of differential expression analysis between 5% and 1% oxygen. It takes in account the intrinsic heterogeneity of glioblastoma, as multiple cell lines were used. This is why, in our study, we were not looking for a single (or few) biomarkers; rather, we focused on a robust signature, which has enough power (despite the variability in gene expression between different glioma samples/patients, due to the contribution of every gene in the signature) to identify patients that could potentially express this hypoxia adaptation. Our gene signature, built using an ANOVA analysis, was valid not only in our GBM lines in vitro, but also in the in silico analyses from GBM transcriptomic data in vivo.

We demonstrated that our hypoxia gene signature correlated with in vivo-generated data (Figure 2a), supporting the use of physioxia at 5% O_2_ as a biologically relevant oxygen condition. Moreover, the signature was also expressed in predicted hypoxic regions from human biopsies documented in GlioVis and Ivy-GAP databases [40,42]. High expression of the signature was correlated with certain immune- and glycolysis-associated genes (Figure 2b,c), and was enriched for gene clusters of hypoxia, glycolysis, and angiogenesis (Figure 1c), consistent with previous studies showing angiogenic and immunologic consequences in response to hypoxia in GBM patients [52]. Furthermore, expression of the signature was highly correlated with poor survival in GBM patients, confirming it to be relevant and robust. Moreover, the low expression of our signature in *IDH*-mutant GBM reinforces earlier observations of *IDH* mutation being associated with HIF-1α inhibition [53,54], although this was not the case with a smaller patient cohort [55].

In our study, we directly modulated oxygen availability in cell cultures, rather than directly modulating the transcription factor HIF-1α, allowing us to study all potential adaptations of GBM cells to oxygen deprivation, without limiting our findings to one transcription factor. Indeed, half of the genes in our signature (Figure S1d,e) are not reported to be direct targets of HIF [47,48]. Several HIF-independent mechanisms have been described, such as mTOR inactivation [19], or the activation of NF-κB through reactive oxygen species (ROS) production [56].

Metformin is a well-characterized inhibitor of gluconeogenesis, but in the past decade there has been accumulating evidence of its anti-cancer effects [57], mainly through the reduction of cancer cell growth. This is consistent with our in vitro results showing a reduced number of viable GBM cells after treatment. Metformin is currently in clinical trials for many cancer types (over 300 registered in clinicaltrials.gov), including GBM. Retrospective studies of high-grade glioma patients taking metformin medication (mainly because of a previous diabetes diagnosis) indicated improved outcomes in relation to anaplastic astrocytomas (grade III gliomas), but not in relation to GBM [58,59], although a statistically nonsignificant association of metformin monotherapy with glioblastoma survival at baseline was reported [59]. Studies in xenografted mice demonstrated that when metformin was used at doses higher than those used for diabetes, there was a survival benefit, together with sensitization to concomitant radio-chemotherapy [26]. Moreover, other studies have confirmed the benefits of combining metformin with temozolomide chemotherapy in vitro and in vivo in mouse models [26,60,61]. This has encouraged clinical development, and several trials are ongoing (ClinicalTrials.gov Identifiers: NCT03243851, NCT02780024, NCT01430351, and NCT02149459). A further potential benefit of metformin treatment is a reduction in GBM cell invasion [62,63], one of the consequences of tumor hypoxia.

Metformin reduces OCR, as we have validated under hyperoxic conditions, and reported for the first time under physiologically-relevant oxygenation. This most likely occurs by inhibiting complex I of the electron transport chain in the mitochondria, causing energetic stress in cells [64]. Metformin is reported to reduce glucose output through the decrease in cAMP, protein kinase A activity, and phosphorylation of protein kinase A substrates [64]. Although the stimulatory effect of metformin on glycolysis is linked to inhibition of complex I, this effect could be weakened by increased glutamine metabolism, consistent with the lack of upregulation of glycolysis-associated genes in our hypoxia signature following metformin treatment (Figure 5). The consequence of these processes push cells to react by rewiring the metabolic flux, reducing oxidative phosphorylation, coupled with the inhibition of oxygen consumption, the latter being directly observed in our in vitro experiments (Figure 4c,d and Figure S3a,b). These metabolic changes can include upregulation of pathways to support increased glycolysis and/or increased utilization of glutamine (or other metabolites) to provide alternative substrates for ATP production [65]. Additionally, metformin effects could also reduce HIF-1α mRNA and protein levels, which impact HIF-dependent pathways under hypoxic conditions [66,67]. Moreover, a direct decrease of hypoxia-induced HIF-1α protein content can occur through HIF degradation [68]. This indicates that metformin could use multiple mechanism to attenuate the hypoxia gene signature (Figure 5), within which there are genes that are HIF-dependent (HRE-containing; Figure S1e) as well as HIF-independent.

Overall, as metformin reduces oxygen consumption, more oxygen could be available for tumor cells, consistent with the observed attenuation of the hypoxia gene signature (Figure 5). The effect on OCR was seen at 5% O_2_ but not at 1% O_2_, probably because under hypoxia the OCR is already very low. A downside of metformin treatment is the consequent increase in lactate production, increasing the risk of acidosis. In our in vitro settings, metformin maintained the same ECAR, except at high concentrations, in accordance with other studies reporting modest acidification [69]. However, another study reported high lactate production following low doses of metformin treatment in GBM cell lines, but this could be circumvented by combining metformin with drugs inhibiting lactate production [70].

One disadvantage of using metformin is that it affects many cellular pathways, for example, through restriction of important substrates required for TCA cycle-dependent biosynthesis [71], as well as less characterized mechanisms [72]. Indeed, our in vitro experimentation with metformin was designed to determine whether it could have an effect on our hypoxia gene signature, rather than to fully define the metabolic pathways. However, despite incomplete mechanistic understanding of this drug, decades of clinical usage confirm its low toxicity, which, based on our tests (Figure 4a, Figure S2b), we can now extend to T cells, suggesting compatibility with future immunotherapies. In contrast, cultures of non-malignant astrocytes did show some sensitivity to metformin, particularly at high doses. Nevertheless, after decades of studies in animal models, and observations in human patients, metformin is considered to have a favorable impact on the central nervous system, with diminished incidence and progression of neurodegenerative diseases [73].

We had anticipated that *TP53* mutational status might affect glioma cell viability after metformin treatment because of *TP53*’s reported effects on the glycolytic pathway [74]. Since metformin forces cancer cells to shift towards glycolysis or other pathways for ATP production, cells with mutated or loss of *TP53* would not be able to adapt to such metabolic switches and would be selectively inhibited or killed by metformin. Although we did observe apoptosis and necrosis induction after metformin treatment, this was not restricted to those lines with mutated *TP53*. GBM cell metabolism is clearly influenced by multiple genes, which may be differentially mutated in the different lines tested, and which may explain how the particular metabolic weakness due to mutated *TP53* in LN229 may be overridden by other characteristics, and conversely, how other genes contribute to metformin sensitivity in *TP53* WT cell lines. The in vitro effects of metformin on GBM cell viability are arguably not the most important role of this compound, in view of the modest effects and the dose used, which would not easily be achieved in vivo, as has been previously discussed [70].

Using a gene signature, instead of analyzing individual genes, allowed us to identify a robust adaptation of GBM cells to hypoxia. This hypoxia gene signature, which strongly correlated with poor survival, could potentially identify patients most likely to benefit from treatment with metformin or other metabolic drugs, assuming that the compounds achieved similar reversal of the signature in vivo as we observed in vitro. Indeed, metformin can be considered a prototypical metabolic drug. Ultimately, however, newer therapeutic compounds with more favorable pharmacokinetics may become available for clinical metabolic targeting [75,76]. Some of the changes we noted for expression of individual genes after metformin treatment did not follow the general trend of downregulation, for example, *DDIT4* and *VEGFA* (Figure 5 and Table S2). Concerning *DDIT4*, this is involved in the cellular stress response and has been reported to be upregulated in the presence of metformin [77], in accordance with our results. We performed extensive testing of *DDIT4*, including assessment of the corresponding protein expression after metformin exposure under different oxygen conditions. Here both upregulation and downregulation of the protein were observed in different cell lines, making definitive conclusions difficult to reach (data not shown). We also modulated *DDIT4* expression by both small interfering (si) RNA and CRISPR/Cas9 in the SB28 mouse GBM cells. The effect on the metformin response was modest (data not shown). Whether this functional consequence was because of the efficiency of the knockdown, or because multiple other genes are important, could not be resolved from these experiments. Concerning *VEGFA*, this was upregulated, but not uniformly for the three GBM lines tested, nor for all oxygen conditions. Indeed, metformin is proposed to globally inhibit angiogenesis, despite a transient stimulation of pro-angiogenic factors [78], suggesting the possible effect of the duration of metformin treatment on the expression of downstream targets. Overall, given the broad effects of metformin on many pathways, experimental modulation of individual genes identified within our hypoxia signature is unlikely to be a productive way forward in understanding the consequences of using this compound. Ultimately, only assessing the impact of metformin alone or in combination with other compounds in vivo will resolve these issues.

## 5. Conclusions

Taken together, our direct manipulation of oxygenation in vitro, including the use of physioxia, has revealed a hypoxia gene signature that recapitulates human GBM observations in vivo (hypoxic localization and inflammatory and glycolytic responses). Moreover, this hypoxia signature is correlated with shorter survival of GBM patients. Using metformin, we reduced GBM cell growth and oxygen consumption, as well as the expression of key genes of the hypoxia gene signature, supporting further investigation of this drug, or new generation compounds, in the context of GBM therapy.

## Figures and Tables

**Table 1:** Hypoxia signature genes and primers used for qPCR analysis.

Gene ID	Forward Primer	Reverse Primer
**ADM**	TGCCCAGACCCTTATTCG	CCGGAGGCCCTGGAAGT
**ALDOC**	ATGCCTCACTCGTACCCAG	TTTCCACCCCAATTTGGCTCA
**ANGPTL4**	GGCTCAGTGGACTTCAACCG	CCGTGATGCTATGCACCTTCT
**ANKRD37**	TTAGGAGAAGCTCCACTACACAA	CACTGGCTACAAGCAGGCT
**ARRDC3**	TGTATTCTAGTGGGGATACCGTC	TCGCATGTCCTCTTGCATGAA
**BHLHE40**	ATCCAGCGGACTTTCGCTC	TAATTGCGCCGATCCTTTCTC
**CA9**	GGATCTACCTACTGTTGAGGCT	CATAGCGCCAATGACTCTGGT
**DDIT4**	TGAGGATGAACACTTGTGTGC	CCAACTGGCTAGGCATCAGC
**EGLN3**	TCCTGCGGATATTTCCAGAGG	GGTTCCTACGATCTGACCAGAA
**HAS2**	CACTGGGACGAAGTGTGGATTA	GCATAGTGTCTGAATCACAAACCTG
**HILPDA**	GCGCTTTTGTCTCCGGGTC	GTAAGCCCTCTAGGGACTCCA
**HK2**	GAGCCACCACTCACCCTACT	CCAGGCATTCGGCAATGTG
**PGK1**	GAACAAGGTTAAAGCCGAGCC	GTGGCAGATTGACTCCTACCA
**NDRG1**	CTCCTGCAAGAGTTTGATGTCC	TCATGCCGATGTCATGGTAGG
**PDK1**	GGATTGCCCATATCACGTCTTT	TCCCGTAACCCTCTAGGGAATA
**SLC2A1**	TCTGGCATCAACGCTGTCTTC	CGATACCGGAGCCAATGGT
**SLC2A3**	TCCACGCTCATGACTGTTTC	GCCTGGTCCAATTTCAAAGA
**STC1**	AGGTGCAGGAAGAGTGCTACA	GACGACCTCAGTGATGGCTT
**TMEM45A**	GCATGGCTTTAACTGGCATGG	CAGCCCAGGAGTTGATTCCA
**VEGFA**	AGGGCAGAATCATCACGAAGT	AGGGTCTCGATTGGATGGCA

**Table 2:** The Cancer Genome Atlas (TCGA) patient characteristics.

	Hypoxia Signature		
Tumor Variables Initial Diagnosis, *n*	Low Expression (*n* = 161)	High Expression (*n* = 367)	*p*-Value
**MGMT gene promoter status, *n***			0.574
Unmethylated	52	125	
Methylated	57	113	
Unknown	52	129	
**IDH gene status, *n***			<0.001
Mutant	23	7	
Wildtype	109	263	
Unknown	29	97	

## Supplementary Materials

The following are available online at https://www.mdpi.com/2079-7737/9/9/264/s1, Figure S1: Whole transcriptome analysis from human GBM cell lines exposed to different oxygen conditions. Figure S2: Functional assays of human GBM cell lines and astrocytes exposed to metformin. Figure S3: Effect of differential oxygen availability and metformin on oxygen consumption rate (OCR) and extracellular acidification rate (ECAR) on human and mouse GBM cell lines. Figure S4: Western blot image and densitometry data for HIF-1α expression by GBM line Ge835 under different oxygen fractions. Table S1: Relative gene expression values used for heat map data in Figure S1c. Table S2: Fold change values of metformin modulation of the hypoxia gene signature used for heat map in Figure 5. Table S3: Univariate and multivariate analysis of the hypoxia signature. Table S4: Survival analysis for all datasets, GBM only. Supplementary File 1: ANOVA analysis used to build hypoxia gene signature. Supplementary File 2: Genes in the hypoxia signature that are differentially expressed in each cell line. Additional file 1: All legends for supplementary data.
