# Supplementary material for: An Experimentally Defined Hypoxia Gene Signature in Glioblastoma and Its Modulation by Metformin
Source: Biology (Basel). 2020 Sep 2;9(9):264. doi: 10.3390/biology9090264 (PMC7563149; doi:10.3390/biology9090264)
Supplement: Supplementary file 1 [file biology-09-00264-s001.zip › CALVO_TARDONetal_Manuscript.docx]

Article

An experimentally defined hypoxia gene signature in glioblastoma and its modulation by metformin

Marta Calvo Tardón^1,#^, Eliana Marinari^1,#^, Denis Migliorini^2^, Viviane Bes^1^, Stoyan Tankov^1^, Emily Charrier^1,a^, Thomas A McKee^3^, Valérie Dutoit^4^, Pierre-Yves Dietrich^4^, Erika Cosset^1,b^ and Paul R Walker^1,^*

^1^ Center for Translational Research in Onco-Hematology, Division of Oncology, Geneva University Hospitals and University of Geneva, 1211 Geneva, Switzerland; (M.C.T) [marta.calvo@unige.ch](mailto:marta.calvo@unige.ch); (E.M.) [eliana.marinari@unige.ch](mailto:eliana.marinari@unige.ch); (V.B.) [viviane.rochemont@unige.ch](mailto:viviane.rochemont@unige.ch); (E.C.^a^) emily.charrier@unige.ch; (E.C.^b^); (S.T.) stoyan.tankov@unige.ch, erika.cosset@unige.ch; (P.R.W.) paul.walker@unige.ch

^2^ Department of Oncology, Clinical Research Unit, Dr Dubois Ferrière Dinu Lipatti Research Foundation, Geneva University Hospitals, 1205 Geneva, Switzerland; dmig@pennmedicine.upenn.edu

^3^ Division of Clinical Pathology, Geneva University Hospitals, 1211 Geneva, Switzerland; [thomas.a.mckee@hcuge.ch](mailto:thomas.a.mckee@hcuge.ch)

^4^ Center for Translational Research in Onco-Hematology, Department of Oncology, Geneva University Hospitals and University of Geneva, 1211 Geneva, Switzerland; (V.D.) [valerie.dutoit@unige.ch](mailto:valerie.dutoit@unige.ch) ; (P.Y.D.) [pierre-yves.dietrich@hcuge.ch](mailto:pierre-yves.dietrich@hcuge.ch)

# Equal contribution

***** Correspondence: Correspondence: [paul.walker@unige.ch](mailto:paul.walker@unige.ch) Tel.: +41 223795079

Received: date; Accepted: date; Published: date

**Abstract:** Glioblastoma multiforme (GBM) is the most common and aggressive primary brain tumor, characterized by a high degree of intertumoral heterogeneity. However, a common feature of the GBM microenvironment is hypoxia, which can promote radio- and chemotherapy resistance, immunosuppression, angiogenesis, and stemness. We experimentally defined common GBM adaptations to physiologically relevant oxygen gradients, and we assessed their modulation by the metabolic drug metformin. We directly exposed human GBM cell lines to hypoxia (1% O_2_) and to physioxia (5% O_2_), we then performed transcriptional profiling, and compared our in vitro findings to predicted hypoxic areas in vivo using in silico analyses. We observed a heterogenous hypoxia response, but also a common gene signature that was induced by a physiologically relevant change in oxygenation from 5% O_2_ to 1% O_2_. In silico analyses showed that this hypoxia signature was highly correlated with a perinecrotic localization in GBM tumors, expression of certain glycolytic and immune-related genes, and poor prognosis of GBM patients. Metformin treatment of GBM cell lines under hypoxia and physioxia reduced viable cell number, oxygen consumption rate, and partially reversed the hypoxia gene signature, supporting further exploration of targeting tumor metabolism as a treatment component for hypoxic GBM.

**Keywords:** Hypoxia; physioxia; glioblastoma; glioblastoma microenvironment; metformin; hypoxia gene signature

1. Introduction

Glioblastoma (GBM) is a diffuse astrocytic tumor, which in the 2016 WHO classification is divided into those that are isocitrate dehydrogenase (*IDH*)-wildtype and those that are *IDH*-mutant [1]. It is the most common and aggressive primary tumor in the central nervous system [2] and median survival of GBM patients is only 12-15 months [3], despite standard of care consisting of surgical resection and radio-chemotherapy. An important characteristic of these tumors is the high level of heterogeneity, both intertumoral [4,5] and intratumoral [6,7].

A common feature found in most solid tumors is the presence of hypoxia as a result of rapid cancer cell proliferation and aberrant vasculature that is unable to maintain oxygen supply [8]. Tumor hypoxia drives malignancy by promoting chemo- and radiotherapy resistance, an immunosuppressive microenvironment, cancer cell stemness, angiogenesis, and metabolic modulation [9-11]. The study of tumor hypoxia in vitro frequently uses cell cultures exposed to atmospheric conditions (21% O_2_) as a control, although this does not represent any physiological oxygen fraction found in vivo [12] and does not always recapitulate cellular functions under physioxia [13]. Physiologic oxygen availability is tissue-dependent, with 2-9% O_2_ (10-40 mmHg) being reported for the healthy brain [14]. Oxygen fractions used to refer to tumor hypoxia vary between studies, but 0.5-2% O_2_ (i.e., less than physiologic values, and thereby inadequate oxygenation) are observed in vivo in the tumor bed and are used experimentally in vitro [15,16]. One of the key regulators of the hypoxia response is hypoxia-inducible factor (HIF)-1α [17], but HIF-independent cellular pathways have also been reported [18,19].

Aberrant signaling pathways such as mTOR, or pro-tumoral functions such as VEGF release have been individually targeted in GBM therapy, using rapamycin (or its derivatives) or bevacizumab, respectively, but with limited impact on overall survival of GBM patients so far [20,21]. Metformin, a type 2 diabetes drug, has been shown to decrease risk of developing certain types of cancer [22], and can potentially target mTOR signaling and also reprogram oxygen metabolism, thereby reducing hypoxia in the tumor microenvironment. Metformin has been shown to improve the anti-tumor immune response in several mouse tumor models [23-25]. In the context of GBM, metformin can inhibit cell growth through mTOR inhibition, and has been observed to enhance the therapeutic effect of temozolomide in human xenografts [26].

In the treatment of GBM, a better understanding of its genetic, epigenetic, and/or transcriptional characteristics could help identify markers or signatures that predict outcome or response to specific therapies, as exemplified by MGMT promoter methylation status that predicts response to temozolomide [27]. More recently, gene mutations and expression profiles are being studied to associate specific gene signatures with clinical outcome [28,29], including hypoxia-induced gene signatures in multiple cancer types [8,30]. Here, we evaluated the GBM response to low levels of oxygen and, despite GBM heterogeneity, we identified a common hypoxia gene signature that was determined experimentally and that was associated with pseudopalisading and necrotic areas of GBM from patient data; the signature correlated with expression of certain glycolysis and immune-related genes, and importantly, survival. We validated the use of metformin to force metabolic changes in GBM cells and to reduce oxygen consumption and numbers of viable tumor cells.

2. Materials and Methods

*2.1. In vitro cultures.*

Human Ge904 at passage (p) 11, Ge835 (p8), and Ge898 (p10) were obtained in house from resection of primary *IDH* wt GBM. Research use of this human material was approved by the local Institutional Review Board and Ethics committee, with signed informed consent obtained for all patients. LN18 (p560) and LN229 (p209) were obtained from ATCC, U87 (unknown passage number), and U251 (p590) were obtained from ECACC; mouse SB28 was kindly provided by H. Okada, UCSF, USA [31]; and GL261-OVA was kindly provided by O. Grauer, University Hospital of Münster, UKM, Germany [32]. Normal human astrocytes were obtained from ScienCell. All cell lines were cultured in serum-containing DMEM-based media, and passaged every 2-3 days. GBM cell lines were exposed to atmospheric O_2_ conditions in a conventional hood and incubator, or to 1% O_2_ or 5% O_2_ using the Ruskinn 300 InVivO2 hypoxia workstation (Baker) for 48h. Media was pre-equilibrated to the desired oxygen level by flushing with the corresponding gas mix. All cell lines were tested as negative for mycoplasma.

All subjects gave their informed consent for inclusion before they participated in the study. The study was conducted in accordance with the Declaration of Helsinki, and the protocol was approved by the Ethics Committees of Geneva University Hospitals and the Canton of Geneva (CCER) (03-126).

*2.2. Sequencing and polymerase chain reaction (PCR).*

Total RNA was extracted using Qiagen RNeasy Kit following manufacturer’s instructions. Gene expression by microarray was employed for Ge835, Ge898, Ge904, LN18, and LN229 using Microarray PrimeView Human Gene Expression Array (Affymetrix); probes and associated analysis files with gene annotation for each set of probes (PrimeView Human Gene Expression Array Library files, version 2014).

qPCR of the hypoxia signature genes was performed to quantify mRNA levels of metformin or vehicle-treated cells exposed to hypoxia or physioxia. Briefly, DNase-treated RNA was used to synthesize cDNA (PrimerScript RT; Takara Bio Inc.) The genes analyzed and the primers used are indicated in Table 1.

**Table 1. Hypoxia signature genes and primers used for qPCR analysis**

| Gene ID | Forward primer | Reverse primer |
| --- | --- | --- |
| ADM | TGCCCAGACCCTTATTCG | CCGGAGGCCCTGGAAGT |
| ALDOC | ATGCCTCACTCGTACCCAG | TTTCCACCCCAATTTGGCTCA |
| ANGPTL4 | GGCTCAGTGGACTTCAACCG | CCGTGATGCTATGCACCTTCT |
| ANKRD37 | TTAGGAGAAGCTCCACTACACAA | CACTGGCTACAAGCAGGCT |
| ARRDC3 | TGTATTCTAGTGGGGATACCGTC | TCGCATGTCCTCTTGCATGAA |
| BHLHE40 | ATCCAGCGGACTTTCGCTC | TAATTGCGCCGATCCTTTCTC |
| CA9 | GGATCTACCTACTGTTGAGGCT | CATAGCGCCAATGACTCTGGT |
| DDIT4 | TGAGGATGAACACTTGTGTGC | CCAACTGGCTAGGCATCAGC |
| EGLN3 | TCCTGCGGATATTTCCAGAGG | GGTTCCTACGATCTGACCAGAA |
| HAS2 | CACTGGGACGAAGTGTGGATTA | GCATAGTGTCTGAATCACAAACCTG |
| HILPDA | GCGCTTTTGTCTCCGGGTC | GTAAGCCCTCTAGGGACTCCA |
| HK2 | GAGCCACCACTCACCCTACT | CCAGGCATTCGGCAATGTG |
| PGK1 | GAACAAGGTTAAAGCCGAGCC | GTGGCAGATTGACTCCTACCA |
| NDRG1 | CTCCTGCAAGAGTTTGATGTCC | TCATGCCGATGTCATGGTAGG |
| PDK1 | GGATTGCCCATATCACGTCTTT | TCCCGTAACCCTCTAGGGAATA |
| SLC2A1 | TCTGGCATCAACGCTGTCTTC | CGATACCGGAGCCAATGGT |
| SLC2A3 | TCCACGCTCATGACTGTTTC | GCCTGGTCCAATTTCAAAGA |
| STC1 | AGGTGCAGGAAGAGTGCTACA | GACGACCTCAGTGATGGCTT |
| TMEM45A | GCATGGCTTTAACTGGCATGG | CAGCCCAGGAGTTGATTCCA |
| VEGFA | AGGGCAGAATCATCACGAAGT | AGGGTCTCGATTGGATGGCA |

2.3. TP53 analysis.

DNA from cell lines Ge898 and Ge904 was sequenced using an Illumina NextSeq 500 instrument using standard protocols. Briefly, libraries were prepared from 100ng of genomic DNA that was fragmented using Kapa hyperplus kit (Roche, Basel CH). TP53 sequences were captured using a custom SureSelect panel (Agilent,Santa Clara CA). Libraries were prepared using an Illumina NextSeq 550/500 v2.5 sequencing reagent kit (Illumina San Diego, CA) and sequencing was performed on an Illumina NextSeq 500 instrument (Illumina San Diego, CA) using a with pair end reads of 150bp. Variants were called using a custom bioinformatics pipeline based on MuTect 2 [33]. The information regarding the TP53 status of the other GBM cell lines was extracted from the literature for Ge835 [34], SB28 [35] and GL261 [36], or from available databases (p53.iarc.fr/CellLines.aspx; and ATCC).

2.4. Western blot

Fifteen μg of whole protein lysates (NP-40 based lysis buffer) or nuclear fractions (NE-PER™ Nuclear and Cytoplasmic Extraction Reagents, ThermoFisher) were loaded onto 12.5% SDS-PAGE gel and transferred onto nitrocellulose membranes. Membranes blocked with 5% non-fat dry milk were incubated with the following antibodies: rabbit anti-HIF-1α (Bethyl), mouse anti-TBP (Novus Biologicals), followed by goat anti-rabbit IgG-HRP (Sigma) or goat anti-mouse IgG-HRP (Sigma). ECL detection (SuperSignal West Pico, ThermoFisher) was used to observe reactive bands.

2.5. In vitro assays

All assays were performed for 48h under the corresponding oxygenation conditions. Viable cell numbers were assessed using CellTiter Glo (Promega), following manufacturer’s protocol, with luminescence measured using a Cytation3 reader (BioTek). Oxygen consumption and extracellular acidification rates were measured using Cell Mito Stress kit (Agilent) in XF media (Agilent) containing 1 g/l glucose, 2 mM glutamine, and 1 mM sodium pyruvate measured in a Seahorse XFe96 Analyzer (Agilent) placed inside a hypoxia station.

2.6. Statistical analysis

The RMA normalized intensities were analyzed for differential expression [37]. The following comparisons were done on each of the 3 independent experiments with a t-test for each cell line and on all samples with a paired sample ANOVA (FC>1.3, p<0.05), using Partek® Genomics Suite® software, version 6.6. The hypoxia gene signature comprised 33 coding genes with Gene Ontology annotation according to the PrimeView Human Gene Expression Array Library used.

In silico analysis included several datasets: for GBM, The Cancer Genome Atlas (TCGA, *n*=528 grade IV) [38] and IvyGAP (*n*=270 grade IV) [39] were used; for high grade glioma including GBM, Rembrandt (*n*=267, of which *n*=79 grade III and *n*=188 grade IV) [36], Phillips (*n*=100, of which *n*=24 grade III and *n*=76 grade IV) [5], Freije (n=85, of which *n*=26 grade III and *n*=59 grade IV) [40], were used. GlioVis data portal was employed for visualization and analysis of brain tumor expression datasets [41]. Analysis was performed in R version 3.3.2 (https://www.R-project.org/) and figures were generated through Morpheus (https://software.broadinstitute.org/morpheus). Gene set enrichment analysis (GSEA) was performed as previously described [42]. TCGA patient characteristics were compared using Chi-Square test by IBM SPSS® statistics, version 25.0.5.

3. Results

We exposed 5 human GBM lines (Ge835, Ge898, Ge904, LN18, and LN229) to various oxygen conditions: inadequate oxygenation (hypoxia, 1% O_2_), physiologic (physioxia, 5% O_2_), and atmospheric (hyperoxia; 21% O_2_) conditions, and performed transcriptional profiling using Affymetrix Microarray. Comparing 1% O_2_ to 5% O_2_ showed an enrichment in the hallmark hypoxia gene set after performing gene set enrichment analysis (GSEA) (Figure 1a). Our experimental approach consisted of directly modulating oxygenation levels, thereby reproducing in vivo attainable oxygen gradients. This allowed us to identify transcriptional changes reported in GSEA [42]. We confirmed hypoxia adaptation by quantifying nuclear stabilization of HIF-1α by western blot (Figure S1a).


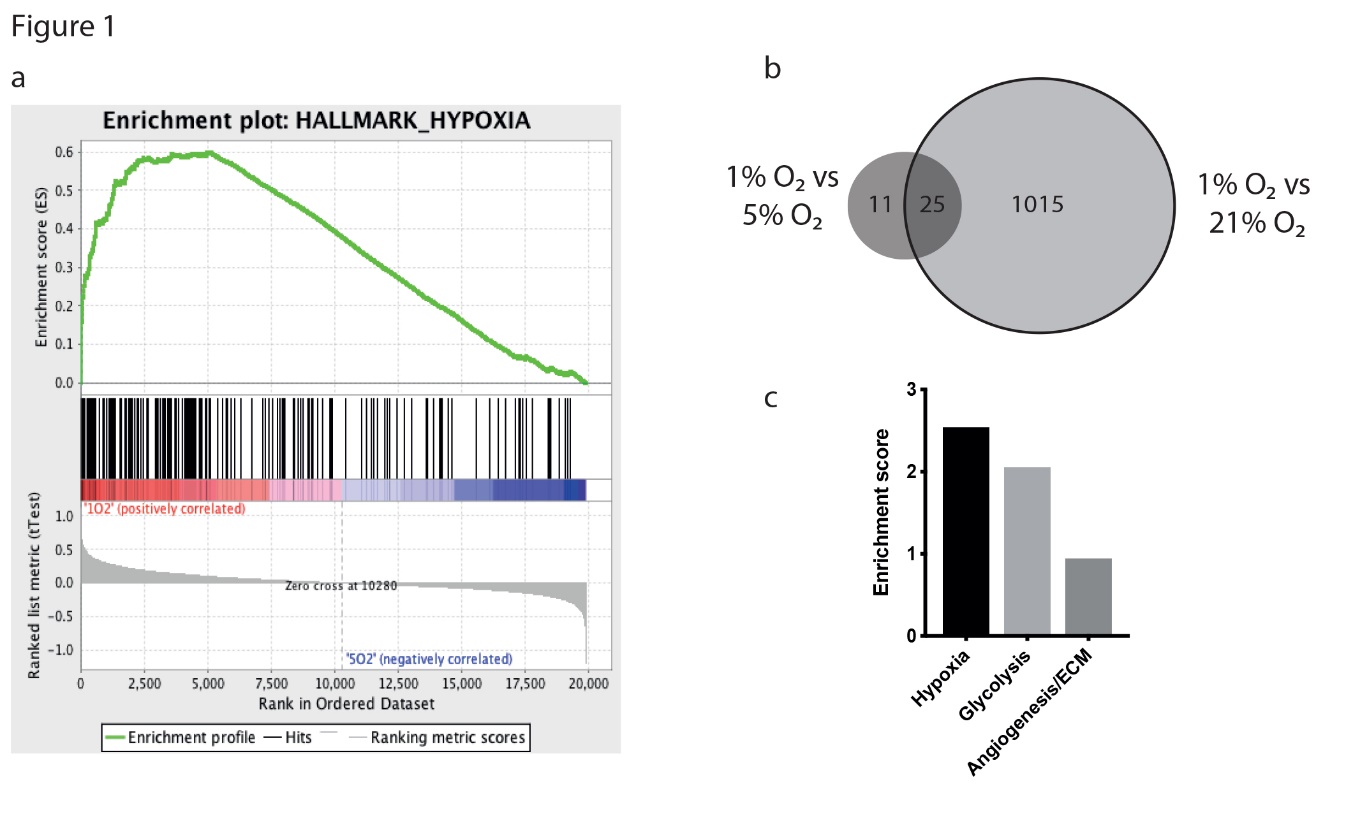


***Figure 1.*** Whole transcriptome analysis of Ge835, Ge898, Ge904, LN18, and LN229 GBM cell lines cultured under hypoxia (1% O_2_), physioxia (5% O_2_), or hyperoxia (21% O_2_) for 48 h. **(a)** Gene set enrichment analysis for hypoxia geneset comparing transcriptional profiles of hypoxia versus physioxia; **(b)** Venn diagram of comparisons between hypoxia and physioxia, and hypoxia and hyperoxia; **(c)** Enrichment scores of gene families from DAVID analysis.

Comparing hypoxia (1% O_2_) to hyperoxia (21% O_2_), we identified 1040 common differentially expressed genes (ANOVA), whereas comparing hypoxia to physioxia (5% O_2_) revealed only 36 differentially expressed genes (Figure S1d). Twenty-five of these 36 genes (69%) were common to the 21% to 1% O_2_ comparison, but 11 genes were unique to the 5% to 1% O_2_ comparison (Figure 1b). This suggests that using atmospheric conditions as a control not only leads to an overestimation of the adaptation of GBM cells to hypoxia, but might also obscure important biological processes taking place under physiologic conditions.

The GBM lines analyzed showed a significant level of heterogeneity. Indeed, unsupervised clustering of the transcriptional data grouped the samples by cell line rather than by the effect of hypoxia (Figure S1c, Table S1). In addition, when different cell lines were separately analyzed for differential expression comparing hypoxia to physioxia, there were no common hits between the 5 cell lines (Figure S1b). Despite this high heterogeneity, when we considered all cell lines together, we could build a hypoxia gene signature based on the common differentially expressed genes with GO annotation between hypoxia and physioxia. Performing DAVID analysis [43], we determined that this experimentally defined signature was significantly enriched for hypoxia, glycolysis, and angiogenesis and extracellular matrix gene clusters (Figure 1c). Of note, 18 of the genes in the signature are not reported to have a hypoxia-responsive element (HRE) sequence [44,45], and therefore may represent HIF-independent hypoxia-regulated responses (Figure S1e).

Interestingly, the signature identified with this experimental approach, directly modulating the availability of oxygen in our GBM cell lines, was highly enriched in predicted hypoxic regions from existing GBM databases. We interrogated our hypoxia signature in the Ivy-GAP platform [39,41], a GBM dataset originating from biopsies and microdissections. The hypoxia signature was highly expressed within perinecrotic and pseudopalisading areas of tumors (predicted to include hypoxic zones) confirming that our signature reflects in vivo observed features (Figure 2a). Our signature was strongly correlated with an inflammatory phenotype that included expression of genes encoding IL-1β, IL-6, and IL-8 (Figure 2b), and with the glycolytic pathway (Figure 2c). GBM (TCGA Table 2) and high-grade glioma patients (from Rembrandt, Phillips, and Freije databases) [5,38,40,46] were clustered according to signature expression, based on k-means cluster analysis (k=2). Importantly, high expression of our signature correlated with poor survival (Figure 3). The TCGA dataset included IDH mutational status; high expression of our signature was strongly correlated with the absence of IDH mutations (Table 2). Moreover, our signature was highly associated with survival in a univariate analysis, but not in a multivariate analysis combined with IDH status, suggesting that it could be an important feature linked to the genetic characteristics of the tumor and not an independent prognostic biomarker (Table S3).

**Table 2.** TCGA patient characteristics.

|  | **Hypoxia Signature** | |  |
| --- | --- | --- | --- |
| **Tumor variables**  **Initial diagnosis, *n*** | **Low expression**  **(*n= 161)*** | **High expression**  **(*n= 367)*** | **p-value** |
| **MGMT gene promoter status, *n*** |  |  | 0.574 |
| Unmethylated | 52 | 125 |  |
| Methylated | 57 | 113 |  |
| Unknown | 52 | 129 |  |
| **IDH gene status, *n*** |  |  | <0.001 |
| Mutant | 23 | 7 |  |
| Wildtype | 109 | 263 |  |
| Unknown | 29 | 97 |  |


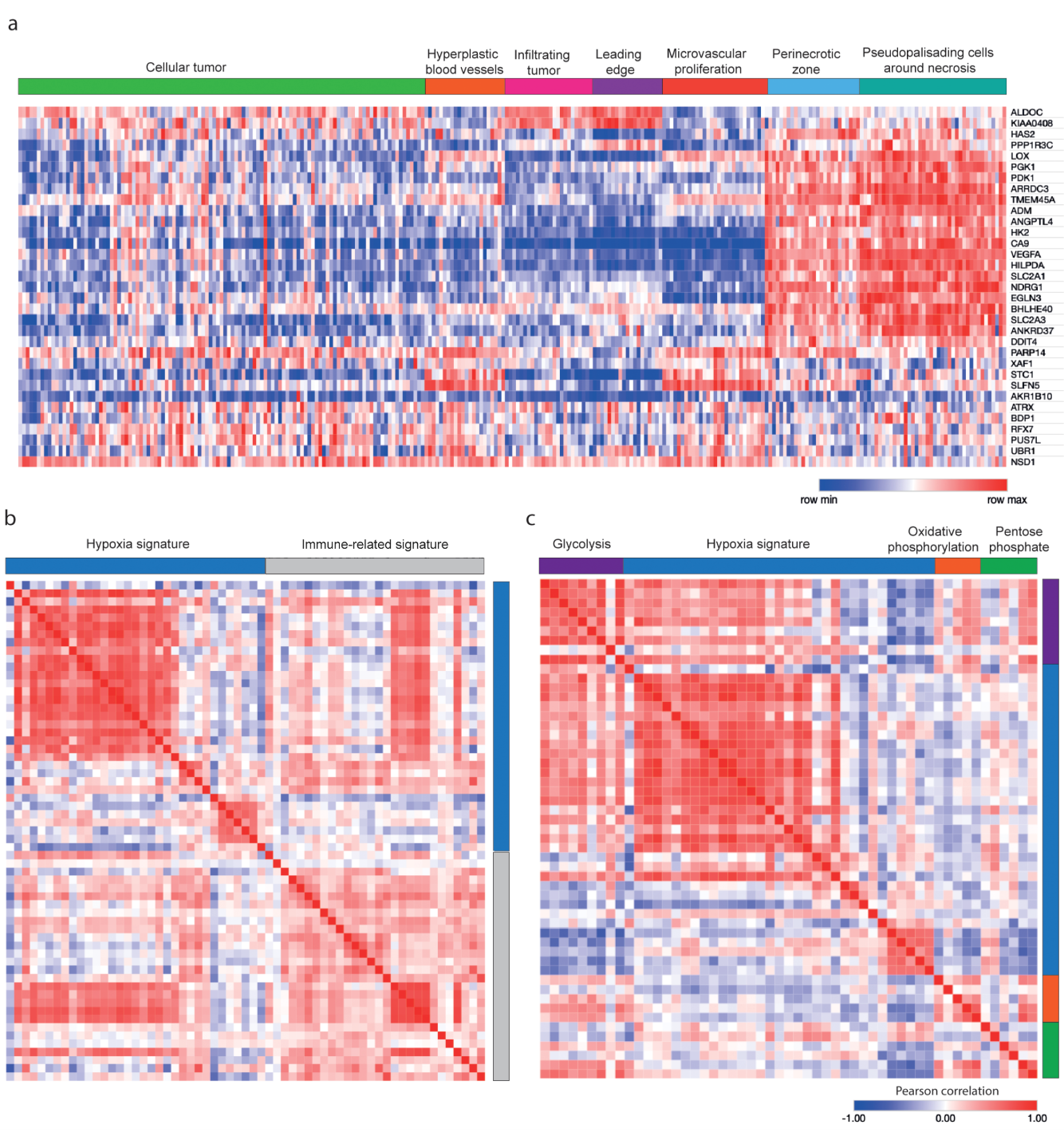


***Figure 2.*** Expression and correlation of the hypoxia gene signature using the Ivy-GAP database. **(a)** Expression of the hypoxia gene signature in different areas of human GBM (n=270) biopsies; **(b,c)** Correlation matrix of the hypoxia signature with **(b)** immune-related genes or **(c)** three metabolic pathways gene lists*.* List of immune-related genes (from left to right and top to bottom): *ARG2, ARG1, TGFB1, IDO1, IL10, CD163, MRC1, CCR4, CCR7, CD80, CD3G, CD3D, CD3E, GZMB, PRF1, CD86, IL1A, IL6, IL1B, IL8, CCL20, TNF, IFNG, IL2, ICAM1, ITGAL, ITGA4, ITGB2.*

Tumor hypoxia could potentially be modulated by the metabolic drug metformin; we therefore investigated its effects on GBM viable cell number and oxygen consumption rate, which were previously only described using non-physiologic oxygen conditions. In these in vitro assays, we used several human GBM cell lines (including Ge835, Ge904, and LN18 for which transcriptional profiling had been performed), and two mouse glioma models (SB28 and GL261 OVA). As expected, metformin reduced the number of viable cells of several of the cell lines in culture (based on ATP quantitation) under physioxia (Figure 4a), and we confirmed that this tendency was maintained under hyperoxia and hypoxia (Figure S2a). There was no significant impact of metformin on normal T cells, but a modest, dose-dependent reduction in number of viable cells from cultures of non-malignant astrocytes (Figure S2b). Since mutational status can impact on metabolism [47], we assessed whether mutations in *PTEN*, *IDH*, and *TP53,* or the promoter methylation status of the gene encoding for the repair enzyme O6-methylguanine-DNA methyltransferase (*MGMT*) impacted on metformin responsiveness. Those cell lines (U251, SB28, GL261 OVA, and LN18) that had a statistically significant reduction in viable cell numbers in response to metformin (p<0.001) were all TP53 mutant, although LN229, which was also *TP53* mutant, showed no sensitivity to metformin in this assay (Figure 4b*)*. Nevertheless, when data from all tested lines was analyzed, response to metformin under physioxia was associated with the *TP53* mutation status (Fischer’s exact test, p<0.05) (Figure 4b). We cannot exclude an importance for mutated *IDH*, as none of the lines harbored *IDH* mutations, however response to metformin under physioxia was not associated with *MGMT* promotor methylation or *PTEN* mutation status (Fischer’s exact test, p>0.05).


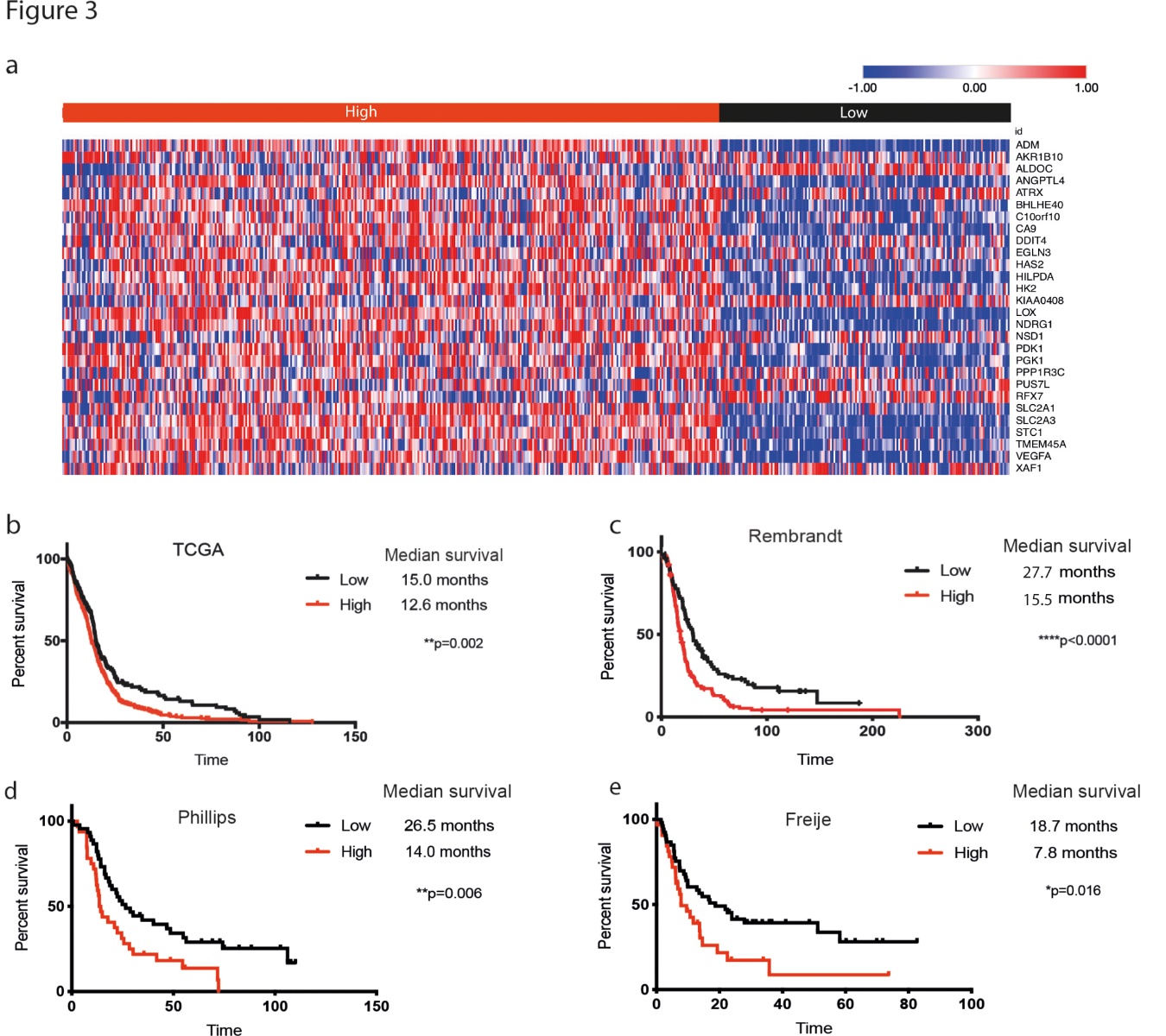


***Figure 3.*** Correlation of the hypoxia signature with survival of high-grade glioma patients. **(a)** Expression of the hypoxia gene signature (z-score) across patients from the TCGA database (n=528), segregated by high and low expression of the signature; **(b-e)** Kaplan-Meier survival curves corresponding with high (red) or low (black) expression of the hypoxia signature in **(b)** TCGA (n=528), **(c)** Rembrandt (n=267), **(d)** Phillips (n=100), and **(e)** Freije (n=85) databases*.*

We evaluated oxygen consumption rate (OCR) and extracellular acidification rate (ECAR) on metformin treated GBM cell lines under hypoxia, physioxia, or hyperoxia. We first validated the reduction on OCR induced by metformin at hyperoxic conditions (Figure 4c; Figure S3a,b). Metformin reduced OCR under physioxia in human GBM cell lines in vitro (Figure 4c; Figure S2a,b). Under hypoxic conditions, the availability of oxygen was clearly a limiting factor in these measurements, indicated by the lower OCR (Figure 4c), which did not permit us to observe significant changes after treatment. Overall, our results suggest that metformin shifted metabolism; this also resulted in a modest trend towards an increased ECAR , but only with high doses, in LN18, Ge904, U251, SB28, and GL261 OVA cell lines (Figure 4d, Figure S2c).


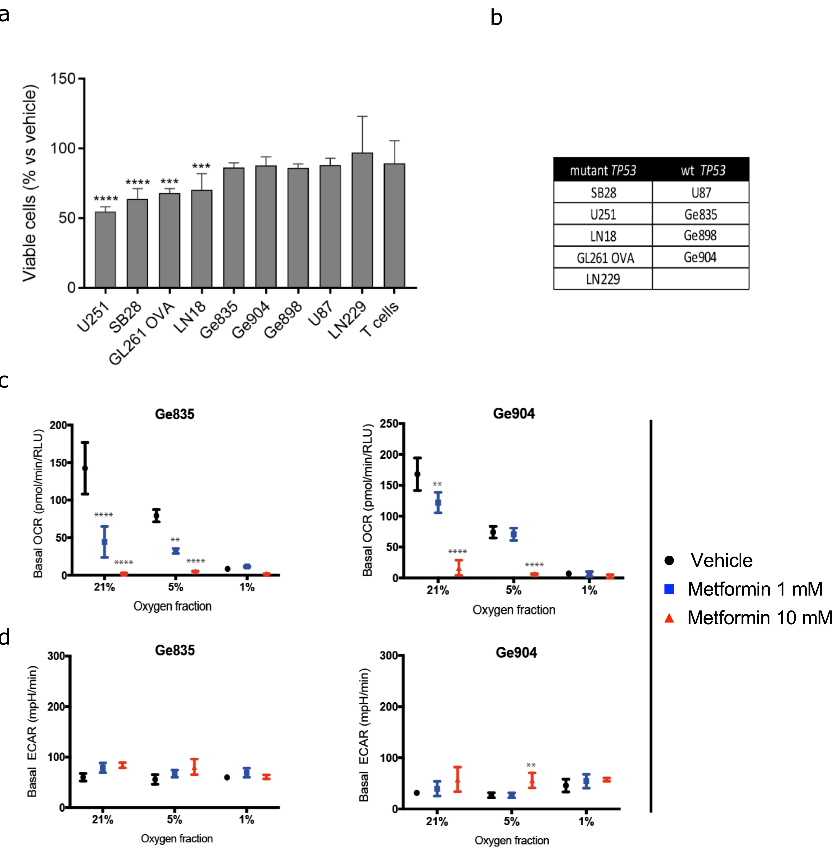


***Figure 4.*** Functional assays of human and mouse GBM cell lines exposed to metformin. **(a)** Effect of 10mM metformin on viable cell number under physioxia; **(b)** Table indicating *TP53* mutation status of all GBM cell lines used; mutant or wild type (wt); **(c)** Basal OCR and **(d)** basal ECAR of Ge835 and Ge904 GBM cell line in vitro at 21%, 5%, and 1% O_2_, with the indicated concentrations of metformin (mean of 3 independent experiments +/- SD; 2-way ANOVA, Sidak’s adjusted p-value. ***p<0.001, ****p<0.0001)*.*

To further assess the consequences of metformin treatment, we tested three of the human GBM lines for which we had both transcriptome data and metabolic analyses (Ge835, LN18, Ge904) and measured expression of our hypoxia signature after metformin treatment. There was a downregulation of most genes of the signature after exposure of these GBM lines to hypoxia or physioxia, although there was a certain level of heterogeneity (Figure 5, Table S2). This effect was more pronounced under hypoxia, compared to physioxia. Some genes, such as *DDIT4* and *VEGFA*, showed instead upregulation by metformin treatment, although this was mostly cell line-specific.


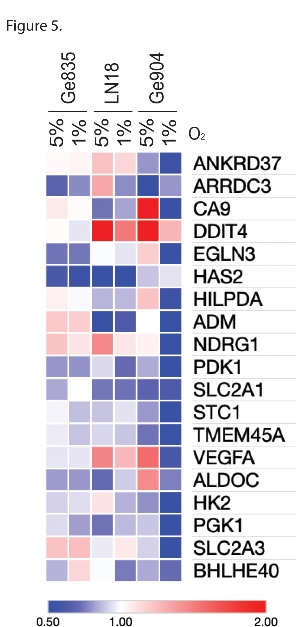


***Figure 5.*** Metformin modulation of the upregulated genes of our hypoxia gene signature. Heat map represents fold-changes of expression between metformin-treated (10mM, 48 h) versus vehicle-treated cells (red, FC>2; blue, FC<0.5). mRNA expression measured by qPCR of upregulated genes from the hypoxia gene signature on Ge835, LN18, and Ge904 treated with metformin or vehicle and exposed to physioxia or hypoxia. Fold change values are tabulated in Table S2.

4. Discussion

Intertumoral heterogeneity of GBM is a known and expected feature. Here we provide detailed evidence of intertumoral heterogeneity at the transcriptional level by performing in vitro hypoxia studies using several human-derived cell lines, which allowed us to identify a robust common hypoxia signature (Figure S1d), despite the heterogeneity. Importantly, we used 5% O_2_ as physiological oxygen control, which more accurately represents the oxygenation levels within the brain and brain tumor tissues. This oxygen fraction has been proven to impact on viable cell number, metabolism, and mitochondrial function [13]. It is probable that changes in gene expression could also occur under other physiologically attainable oxygen fractions, which could be modelled by culture at between 5% and 21% O_2_, and which could, for example, be controlled by different isoforms of HIF that differ in their stability under oxygen in a time and concentration dependent manner [48]. Thus, a hypoxia response can be considered as a spectrum of adaptations that we have sampled at certain points in our study. Nevertheless, we demonstrated that our gene signature correlated with in vivo generated data (Figure 2a), supporting the use of physioxia at 5% O_2_ as a biologically relevant oxygen condition.

The experimentally identified hypoxia signature was found in predicted hypoxic regions from human biopsies documented in GlioVis and Ivy-GAP databases [39,41], highlighting its value and utility for in vivo analyses. This experimental approach adds value to these GBM databases, since we have directly modulated the availability of oxygen in our GBM cell lines, assessing the direct implications of low oxygenation on the glioma cell compartment. High expression of the signature was correlated with certain immune and glycolysis associated genes (Figure 2b,c), and was enriched for gene clusters of hypoxia, glycolysis, and angiogenesis (Figure 1c), consistent with previous studies showing angiogenic and immunologic consequences in response to hypoxia in GBM patients [49]. Furthermore, expression of the signature was highly correlated with poor survival in GBM patients, confirming the importance and robustness of this signature. Moreover, in accordance with these analyses, the low expression of our signature in *IDH* mutant GBM reinforces earlier observations of *IDH* mutation being associated with HIF-1α inhibition [50,51], although this was not the case with a smaller patient cohort [52].

In our study, we directly modulated oxygen availability in cell cultures, rather than directly modulating the transcription factor HIF-1α. This allowed us to study all potential adaptations of GBM cells to lower than physiologic oxygenation, without limiting our findings to one transcription factor. Indeed, half of the genes in our signature (Figure S1d,e) are not reported to be direct targets of HIF [44,45]. Several HIF-independent mechanisms have been described, such as mTOR inactivation [19], or the activation of NF-κB through ROS production [53].

Metformin is a well characterized inhibitor of gluconeogenesis, but in the past decade, there has been accumulating evidence of its anti-cancer effects [54], mainly through the reduction of cancer cell growth, consistent with our in vitro results showing a reduced number of viable GBM cells after treatment. Metformin is currently in clinical trial for many cancer types (over 300 registered in clinicaltrials.gov), including GBM. Retrospective studies of high-grade glioma patients taking metformin medication (mainly because of previous diabetes diagnosis) indicated improved outcome of anaplastic astrocytomas (grade III gliomas), but not of GBM [55,56], although a statistically nonsignificant association of metformin monotherapy with glioblastoma survival at baseline was reported [56]. Studies in xenografted mice demonstrated that when metformin was used at doses higher than those used for diabetes, there was a survival benefit, together with sensitization to concomitant radio-chemotherapy [26]. Moreover, other studies have confirmed benefits of combining metformin with temozolomide chemotherapy in vitro and in vivo in mouse models [26,57,58]. This has encouraged clinical development and several trials are ongoing (ClinicalTrials.gov Identifiers: NCT03243851, NCT02780024, NCT01430351, NCT02149459). A further potential benefit of metformin treatment is a reduction in GBM cell invasion [59,60], one of the consequences of tumor hypoxia.

Metformin reduces OCR, as we have validated under hyperoxic conditions, and reported for the first time under physiologically relevant oxygenation; this is most likely by inhibiting complex I of the electron transport chain in the mitochondria, causing energetic stress in cells [40]. The most explored mechanism of action of metformin is the reduction of glucose output through the decrease in cAMP, protein kinase A activity and phosphorylation of protein kinase A substrates [61]. Although the stimulatory effect of metformin on glycolysis is linked with its ability to inhibit complex I, this effect could be weakened by increased glutamine metabolism, which could explain our observation that the glycolysis-associated genes in our hypoxia gene signature were not upregulated following metformin treatment (Figure 5). The consequence of these processes push cells to react by rewiring the metabolic flux, reducing oxidative phosphorylation coupled with the inhibition of oxygen consumption, the latter being directly observed in our in vitro experiments (Figure 4c,d, Figure S3a,b). These metabolic changes can include upregulation of pathways to support increased glycolysis and/or increased glutamine utilization (or other metabolites) to provide alternative substrates for ATP production [62]. Additionally, metformin effects could also reduce HIF-1α mRNA and protein levels that impact HIF-dependent pathways under hypoxic conditions [63,64]. Moreover, a direct decrease of hypoxia-induced HIF-1α protein content can occur through HIF degradation [65]. This indicates that metformin could use multiple mechanism to attenuate the hypoxia gene signature (Figure 5), within which there are genes that are HIF-dependent (HRE containing; Figure S1e) as well as HIF-independent.

Overall, as less oxygen is being consumed, more oxygen could be available in the tumor microenvironment and in the cell cytoplasm, thus reducing tumor hypoxia and hypoxia-associated responses, as shown by the attenuation of the hypoxia gene signature (Figure 5). We observed an effect on OCR at 5% O_2_ but not at 1% O_2_, probably because under hypoxia the OCR is already very low. Extrapolating to in vivo use of the compound, this could potentially limit expansion of hypoxic regions, encouraging the use of metformin at early stages where hypoxic regions are less extensive. A downside of metformin treatment is the consequent increase in lactate production, increasing the risk of acidosis. In our in vitro settings, metformin maintained the same ECAR, except at high concentration, in accordance with other studies reporting modest acidification [66]. However, another study reported high lactate production following low doses of metformin treatment in GBM cell lines, but this could be circumvented by combining metformin with drugs inhibiting lactate production [67].

One disadvantage of using metformin is that it affects many cellular pathways, for example, through restriction of important substrates required for TCA cycle-dependent biosynthesis[68], as well as less characterized mechanisms [69]. Indeed, our in vitro experimentation with metformin was designed to determine whether it could impact on our hypoxia gene signature, rather than to fully define the metabolic pathways by which these changes occurred. However, despite incomplete mechanistic understanding of this drug, decades of clinical usage confirm its low toxicity, which, based on our tests (Figures 4a, S2b), we can now extend to T cells, suggesting compatibility with future immunotherapies. In contrast, cultures of non-malignant astrocytes did show some sensitivity metformin, particularly at high doses. Nevertheless, after decades of studies in animal models, and observations in human patients, metformin is considered to have a favorable impact on the central nervous system, with diminished incidence and progression of neurodegenerative diseases [70].

We could segregate all GBM cell lines tested into two groups depending on the presence or absence of response to metformin; this showed a statistical correlation with mutated or wt *TP53* status, respectively (Figure 4b). *TP53* has been reported to affect the glycolytic pathway [71]. Since metformin forces cancer cells to shift towards glycolysis or other pathways for ATP production, cells with mutated or loss of *TP53* cannot adapt to such metabolic switchs and are selectively inhibited or killed by metformin. However, GBM cell metabolism is clearly influenced by multiple genes which may be differentially mutated in the different lines tested, which may explain how a particular metabolic weakness due to mutated *TP53* in LN229 may be overridden by characteristics of this particular cell line. The in vitro effects of metformin on GBM cell viability are arguably not the most important role of this compound, in view of the modest effects and the dose used, which would not easily be achieved in vivo, as has been previously discussed [67].

Using a gene signature instead of analyzing individual genes allowed us to identify a robust adaptation of GBM cells to hypoxia. This hypoxia gene signature, which strongly correlated with poor survival, could potentially identify patients most likely to benefit from treatment with metformin or other metabolic drugs, assuming that the compounds achieved similar reversal of the signature in vivo as we observed in vitro with metformin-treated GBM cell lines. Indeed, metformin can be considered as a prototypic metabolic drug, but ultimately, newer therapeutic compounds with more favorable pharmacokinetics may become available for clinical metabolic targeting [72,73]. Some of the changes we noted for expression of individual genes after metformin treatment were not following the general trend of downregulation, for example, *DDIT4* and *VEGFA* (Figure 5 and Table S2*)*. Concerning *DDIT4*, this is involved in the cellular stress response and has been reported to be upregulated in the presence of metformin [74], in accordance with our results. We performed extensive testing of *DDIT4,* including assessment of the corresponding protein expression after metformin exposure under different oxygen conditions; here both upregulation and downregulation of protein were observed in different cell lines, making definitive conclusions difficult to reach (data not shown). We also modulated *DDIT4* expression by both siRNA and CRISPR/Cas9 in the SB28 mouse GBM cells; the impact on the metformin response was modest (data not shown). Whether this functional consequence was because of the efficiency of the knockdown, or because multiple other genes are important could not be resolved from these experiments. Concerning *VEGFA*, this was upregulated in some of the cell lines, although this was not uniform for the three GBM lines tested, nor for all oxygen conditions. Indeed, metformin is proposed to globally inhibit angiogenesis, despite a transient stimulation of pro-angiogenic factors [75], suggesting a possible effect of the duration of metformin treatment on the expression of downstream targets. Overall, given the broad effects of metformin on many pathways, experimental modulation of individual genes identified within our hypoxia signature is unlikely to be a productive way forward in understanding the consequences of using this compound. Ultimately, only assessing the impact of metformin alone or in combination with other compounds in vivo will resolve these issues.

5. Conclusions

Taken together, our direct manipulation of oxygenation in vitro, including use of physioxia, has revealed a hypoxia gene signature that recapitulates human GBM observations in vivo (hypoxic localization, and inflammatory and glycolytic responses). Moreover, this hypoxia signature is correlated with shorter survival of GBM patients. Using metformin, we reduced GBM cell growth and oxygen consumption, as well as expression of key genes of the hypoxia gene signature, supporting further investigation of this drug, or new generation compounds, in the context of GBM therapy.

**Supplementary Materials:** The following are available online at www.mdpi.com/xxx/s1, Figure S1: Whole transcriptome analysis from human GBM cell lines exposed to different oxygen conditions. Figure S2: Functional assays of human GBM cell lines and astrocytes exposed to metformin. Figure S3: Effect of differential oxygen availability and metformin on oxygen consumption rate (OCR) and extracellular acidification rate (ECAR) on human and mouse GBM cell lines. Figure S4: Western blot image and densitometry data for HIF-1α expression by GBM line Ge835 under different oxygen fractions. Table S1. Relative gene expression values used for heat map data in Figure S1c. Table S2. Fold change values of metformin modulation of the hypoxia gene signature used for heat map in Figure 5. Table S3. Univariate and multivariate analysis of the hypoxia signature.

**Author Contributions:** Conceptualization, Marta Calvo Tardón, Eliana Marinari, Denis Migliorini, Emily Charrier, Erika Cosset and Paul R Walker; Formal analysis, Marta Calvo Tardón, Eliana Marinari Thomas A McKee, and Stoyan Tankov; Funding acquisition, Pierre-Yves Dietrich and Paul R Walker; Investigation, Marta Calvo Tardón, Viviane Bes, Emily Charrier, Thomas A McKee, Erika Cosset and Stoyan Tankov; Supervision, Paul R Walker; Writing – original draft, Marta Calvo Tardón, Eliana Marinari and Paul R Walker; Writing – review & editing, Marta Calvo Tardón, Eliana Marinari, Denis Migliorini, Valérie Dutoit, Pierre-Yves Dietrich, Erika Cosset, Stoyan Tankov and Paul R Walker.

**Funding:** This work was funded by Fondation Medic, the Association Frédéric Fellay, and Fond’action contre le cancer.

**Acknowledgments:** We thank Géraldine Philippin for assistance with cell culture.

**Conflicts of Interest:** The authors declare no conflict of interest. The funders had no role in the design of the study; in the collection, analyses, or interpretation of data; in the writing of the manuscript, or in the decision to publish the results.

**References**

1. Louis, D.N.; Perry, A.; Reifenberger, G.; von Deimling, A.; Figarella-Branger, D.; Cavenee, W.K.; Ohgaki, H.; Wiestler, O.D.; Kleihues, P.; Ellison, D.W. The 2016 World Health Organization Classification of Tumors of the Central Nervous System: a summary. *Acta neuropathologica* **2016**, *131*, 803-820, doi:10.1007/s00401-016-1545-1.

2. Wen, P.Y.; Kesari, S. Malignant gliomas in adults. *N Engl J Med* **2008**, *359*, 492-507, doi:10.1056/NEJMra0708126.

3. Stupp, R.; Mason, W.P.; van den Bent, M.J.; Weller, M.; Fisher, B.; Taphoorn, M.J.; Belanger, K.; Brandes, A.A.; Marosi, C.; Bogdahn, U., et al. Radiotherapy plus concomitant and adjuvant temozolomide for glioblastoma. *N Engl J Med* **2005**, *352*, 987-996, doi:10.1056/NEJMoa043330.

4. Verhaak, R.G.; Hoadley, K.A.; Purdom, E.; Wang, V.; Qi, Y.; Wilkerson, M.D.; Miller, C.R.; Ding, L.; Golub, T.; Mesirov, J.P., et al. Integrated genomic analysis identifies clinically relevant subtypes of glioblastoma characterized by abnormalities in PDGFRA, IDH1, EGFR, and NF1. *Cancer Cell* **2010**, *17*, 98-110, doi:10.1016/j.ccr.2009.12.020.

5. Phillips, H.S.; Kharbanda, S.; Chen, R.; Forrest, W.F.; Soriano, R.H.; Wu, T.D.; Misra, A.; Nigro, J.M.; Colman, H.; Soroceanu, L., et al. Molecular subclasses of high-grade glioma predict prognosis, delineate a pattern of disease progression, and resemble stages in neurogenesis. *Cancer Cell* **2006**, *9*, 157-173, doi:10.1016/j.ccr.2006.02.019.

6. Sottoriva, A.; Spiteri, I.; Piccirillo, S.G.; Touloumis, A.; Collins, V.P.; Marioni, J.C.; Curtis, C.; Watts, C.; Tavare, S. Intratumor heterogeneity in human glioblastoma reflects cancer evolutionary dynamics. *Proc Natl Acad Sci U S A* **2013**, *110*, 4009-4014, doi:10.1073/pnas.1219747110.

7. Patel, A.P.; Tirosh, I.; Trombetta, J.J.; Shalek, A.K.; Gillespie, S.M.; Wakimoto, H.; Cahill, D.P.; Nahed, B.V.; Curry, W.T.; Martuza, R.L., et al. Single-cell RNA-seq highlights intratumoral heterogeneity in primary glioblastoma. *Science* **2014**, *344*, 1396-1401, doi:10.1126/science.1254257.

8. Bhandari, V.; Hoey, C.; Liu, L.Y.; Lalonde, E.; Ray, J.; Livingstone, J.; Lesurf, R.; Shiah, Y.J.; Vujcic, T.; Huang, X., et al. Molecular landmarks of tumor hypoxia across cancer types. *Nat Genet* **2019**, *51*, 308-318, doi:10.1038/s41588-018-0318-2.

9. Tredan, O.; Galmarini, C.M.; Patel, K.; Tannock, I.F. Drug resistance and the solid tumor microenvironment. *J Natl Cancer Inst* **2007**, *99*, 1441-1454, doi:10.1093/jnci/djm135.

10. Vaupel, P. Hypoxia and aggressive tumor phenotype: implications for therapy and prognosis. *Oncologist* **2008**, *13 Suppl 3*, 21-26, doi:10.1634/theoncologist.13-S3-21.

11. Wei, J.; Wu, A.; Kong, L.Y.; Wang, Y.; Fuller, G.; Fokt, I.; Melillo, G.; Priebe, W.; Heimberger, A.B. Hypoxia potentiates glioma-mediated immunosuppression. *PLoS One* **2011**, *6*, e16195, doi:10.1371/journal.pone.0016195.

12. Keeley, T.P.; Mann, G.E. Defining Physiological Normoxia for Improved Translation of Cell Physiology to Animal Models and Humans. *Physiol Rev* **2019**, *99*, 161-234, doi:10.1152/physrev.00041.2017.

13. Timpano, S.; Guild, B.D.; Specker, E.J.; Melanson, G.; Medeiros, P.J.; Sproul, S.L.J.; Uniacke, J. Physioxic human cell culture improves viability, metabolism, and mitochondrial morphology while reducing DNA damage. *FASEB J* **2019**, *33*, 5716-5728, doi:10.1096/fj.201802279R.

14. Erecinska, M.; Silver, I.A. Tissue oxygen tension and brain sensitivity to hypoxia. *Respir Physiol* **2001**, *128*, 263-276.

15. Vuillefroy de Silly, R.; Ducimetiere, L.; Yacoub Maroun, C.; Dietrich, P.Y.; Derouazi, M.; Walker, P.R. Phenotypic switch of CD8(+) T cells reactivated under hypoxia toward IL-10 secreting, poorly proliferative effector cells. *Eur J Immunol* **2015**, *45*, 2263-2275, doi:10.1002/eji.201445284.

16. Vaupel, P.; Hockel, M.; Mayer, A. Detection and characterization of tumor hypoxia using pO2 histography. *Antioxid Redox Signal* **2007**, *9*, 1221-1235, doi:10.1089/ars.2007.1628.

17. Iyer, N.V.; Kotch, L.E.; Agani, F.; Leung, S.W.; Laughner, E.; Wenger, R.H.; Gassmann, M.; Gearhart, J.D.; Lawler, A.M.; Yu, A.Y., et al. Cellular and developmental control of O2 homeostasis by hypoxia-inducible factor 1 alpha. *Genes Dev* **1998**, *12*, 149-162, doi:10.1101/gad.12.2.149.

18. Park, E.C.; Ghose, P.; Shao, Z.; Ye, Q.; Kang, L.; Xu, X.Z.; Powell-Coffman, J.A.; Rongo, C. Hypoxia regulates glutamate receptor trafficking through an HIF-independent mechanism. *EMBO J* **2012**, *31*, 1379-1393, doi:10.1038/emboj.2011.499.

19. Arsham, A.M.; Howell, J.J.; Simon, M.C. A novel hypoxia-inducible factor-independent hypoxic response regulating mammalian target of rapamycin and its targets. *The Journal of biological chemistry* **2003**, *278*, 29655-29660, doi:10.1074/jbc.M212770200.

20. Akhavan, D.; Cloughesy, T.F.; Mischel, P.S. mTOR signaling in glioblastoma: lessons learned from bench to bedside. *Neuro Oncol* **2010**, *12*, 882-889, doi:10.1093/neuonc/noq052.

21. Wick, W.; Gorlia, T.; Bendszus, M.; Taphoorn, M.; Sahm, F.; Harting, I.; Brandes, A.A.; Taal, W.; Domont, J.; Idbaih, A., et al. Lomustine and Bevacizumab in Progressive Glioblastoma. *N Engl J Med* **2017**, *377*, 1954-1963, doi:10.1056/NEJMoa1707358.

22. Bowker, S.L.; Majumdar, S.R.; Veugelers, P.; Johnson, J.A. Increased cancer-related mortality for patients with type 2 diabetes who use sulfonylureas or insulin. *Diabetes Care* **2006**, *29*, 254-258, doi:10.2337/diacare.29.02.06.dc05-1558.

23. Ding, L.; Liang, G.; Yao, Z.; Zhang, J.; Liu, R.; Chen, H.; Zhou, Y.; Wu, H.; Yang, B.; He, Q. Metformin prevents cancer metastasis by inhibiting M2-like polarization of tumor associated macrophages. *Oncotarget* **2015**, *6*, 36441-36455, doi:10.18632/oncotarget.5541.

24. Eikawa, S.; Nishida, M.; Mizukami, S.; Yamazaki, C.; Nakayama, E.; Udono, H. Immune-mediated antitumor effect by type 2 diabetes drug, metformin. *Proc Natl Acad Sci U S A* **2015**, *112*, 1809-1814, doi:10.1073/pnas.1417636112.

25. Scharping, N.E.; Menk, A.V.; Whetstone, R.D.; Zeng, X.; Delgoffe, G.M. Efficacy of PD-1 Blockade Is Potentiated by Metformin-Induced Reduction of Tumor Hypoxia. *Cancer Immunol Res* **2017**, *5*, 9-16, doi:10.1158/2326-6066.CIR-16-0103.

26. Sesen, J.; Dahan, P.; Scotland, S.J.; Saland, E.; Dang, V.T.; Lemarie, A.; Tyler, B.M.; Brem, H.; Toulas, C.; Cohen-Jonathan Moyal, E., et al. Metformin inhibits growth of human glioblastoma cells and enhances therapeutic response. *PLoS One* **2015**, *10*, e0123721, doi:10.1371/journal.pone.0123721.

27. Hegi, M.E.; Diserens, A.C.; Gorlia, T.; Hamou, M.F.; de Tribolet, N.; Weller, M.; Kros, J.M.; Hainfellner, J.A.; Mason, W.; Mariani, L., et al. MGMT gene silencing and benefit from temozolomide in glioblastoma. *N Engl J Med* **2005**, *352*, 997-1003, doi:10.1056/NEJMoa043331.

28. Ceccarelli, M.; Barthel, F.P.; Malta, T.M.; Sabedot, T.S.; Salama, S.R.; Murray, B.A.; Morozova, O.; Newton, Y.; Radenbaugh, A.; Pagnotta, S.M., et al. Molecular Profiling Reveals Biologically Discrete Subsets and Pathways of Progression in Diffuse Glioma. *Cell* **2016**, *164*, 550-563, doi:10.1016/j.cell.2015.12.028.

29. Gravendeel, L.A.; Kouwenhoven, M.C.; Gevaert, O.; de Rooi, J.J.; Stubbs, A.P.; Duijm, J.E.; Daemen, A.; Bleeker, F.E.; Bralten, L.B.; Kloosterhof, N.K., et al. Intrinsic gene expression profiles of gliomas are a better predictor of survival than histology. *Cancer Res* **2009**, *69*, 9065-9072, doi:10.1158/0008-5472.CAN-09-2307.

30. Chang, W.H.; Forde, D.; Lai, A.G. A novel signature derived from immunoregulatory and hypoxia genes predicts prognosis in liver and five other cancers. *J Transl Med* **2019**, *17*, 14, doi:10.1186/s12967-019-1775-9.

31. Genoud, V.; Marinari, E.; Nikolaev, S.I.; Castle, J.C.; Bukur, V.; Dietrich, P.Y.; Okada, H.; Walker, P.R. Responsiveness to anti-PD-1 and anti-CTLA-4 immune checkpoint blockade in SB28 and GL261 mouse glioma models. *Oncoimmunology* **2018**, *7*, e1501137, doi:10.1080/2162402X.2018.1501137.

32. Grauer, O.M.; Molling, J.W.; Bennink, E.; Toonen, L.W.; Sutmuller, R.P.; Nierkens, S.; Adema, G.J. TLR ligands in the local treatment of established intracerebral murine gliomas. *J Immunol* **2008**, *181*, 6720-6729, doi:10.4049/jimmunol.181.10.6720.

33. Cibulskis, K.; Lawrence, M.S.; Carter, S.L.; Sivachenko, A.; Jaffe, D.; Sougnez, C.; Gabriel, S.; Meyerson, M.; Lander, E.S.; Getz, G. Sensitive detection of somatic point mutations in impure and heterogeneous cancer samples. *Nat Biotechnol* **2013**, *31*, 213-219, doi:10.1038/nbt.2514.

34. Cosset, E.; Ilmjarv, S.; Dutoit, V.; Elliott, K.; von Schalscha, T.; Camargo, M.F.; Reiss, A.; Moroishi, T.; Seguin, L.; Gomez, G., et al. Glut3 Addiction Is a Druggable Vulnerability for a Molecularly Defined Subpopulation of Glioblastoma. *Cancer Cell* **2017**, *32*, 856-868 e855, doi:10.1016/j.ccell.2017.10.016.

35. Kosaka, A.; Ohkuri, T.; Okada, H. Combination of an agonistic anti-CD40 monoclonal antibody and the COX-2 inhibitor celecoxib induces anti-glioma effects by promotion of type-1 immunity in myeloid cells and T-cells. *Cancer Immunol Immunother* **2014**, *63*, 847-857, doi:10.1007/s00262-014-1561-8.

36. Szatmari, T.; Lumniczky, K.; Desaknai, S.; Trajcevski, S.; Hidvegi, E.J.; Hamada, H.; Safrany, G. Detailed characterization of the mouse glioma 261 tumor model for experimental glioblastoma therapy. *Cancer Sci* **2006**, *97*, 546-553, doi:10.1111/j.1349-7006.2006.00208.x.

37. Irizarry, R.A.; Bolstad, B.M.; Collin, F.; Cope, L.M.; Hobbs, B.; Speed, T.P. Summaries of Affymetrix GeneChip probe level data. *Nucleic acids research* **2003**, *31*, e15, doi:10.1093/nar/gng015.

38. Atlas, T.C.G. Available online:(accessed on 1 November 2015). **2015**.

39. Puchalski, R.B.; Shah, N.; Miller, J.; Dalley, R.; Nomura, S.R.; Yoon, J.G.; Smith, K.A.; Lankerovich, M.; Bertagnolli, D.; Bickley, K., et al. An anatomic transcriptional atlas of human glioblastoma. *Science* **2018**, *360*, 660-663, doi:10.1126/science.aaf2666.

40. Freije, W.A.; Castro-Vargas, F.E.; Fang, Z.; Horvath, S.; Cloughesy, T.; Liau, L.M.; Mischel, P.S.; Nelson, S.F. Gene expression profiling of gliomas strongly predicts survival. *Cancer Res* **2004**, *64*, 6503-6510, doi:10.1158/0008-5472.CAN-04-0452.

41. Bowman, R.L.; Wang, Q.; Carro, A.; Verhaak, R.G.; Squatrito, M. GlioVis data portal for visualization and analysis of brain tumor expression datasets. *Neuro Oncol* **2017**, *19*, 139-141, doi:10.1093/neuonc/now247.

42. Subramanian, A.; Tamayo, P.; Mootha, V.K.; Mukherjee, S.; Ebert, B.L.; Gillette, M.A.; Paulovich, A.; Pomeroy, S.L.; Golub, T.R.; Lander, E.S., et al. Gene set enrichment analysis: a knowledge-based approach for interpreting genome-wide expression profiles. *Proc Natl Acad Sci U S A* **2005**, *102*, 15545-15550, doi:10.1073/pnas.0506580102.

43. Huang da, W.; Sherman, B.T.; Lempicki, R.A. Systematic and integrative analysis of large gene lists using DAVID bioinformatics resources. *Nat Protoc* **2009**, *4*, 44-57, doi:10.1038/nprot.2008.211.

44. Manalo, D.J.; Rowan, A.; Lavoie, T.; Natarajan, L.; Kelly, B.D.; Ye, S.Q.; Garcia, J.G.; Semenza, G.L. Transcriptional regulation of vascular endothelial cell responses to hypoxia by HIF-1. *Blood* **2005**, *105*, 659-669, doi:10.1182/blood-2004-07-2958.

45. Schodel, J.; Oikonomopoulos, S.; Ragoussis, J.; Pugh, C.W.; Ratcliffe, P.J.; Mole, D.R. High-resolution genome-wide mapping of HIF-binding sites by ChIP-seq. *Blood* **2011**, *117*, e207-217, doi:10.1182/blood-2010-10-314427.

46. Scarpace, L.F., E.; Jain, R.; Mikkelsen, T.; Andrews, D. W. Data From REMBRANDT. In *The Cancer Imaging Archive.*, 2015.

47. DeBerardinis, R.J.; Chandel, N.S. Fundamentals of cancer metabolism. *Sci Adv* **2016**, *2*, e1600200, doi:10.1126/sciadv.1600200.

48. Koh, M.Y.; Powis, G. Passing the baton: the HIF switch. *Trends Biochem Sci* **2012**, *37*, 364-372, doi:10.1016/j.tibs.2012.06.004.

49. Murat, A.; Migliavacca, E.; Hussain, S.F.; Heimberger, A.B.; Desbaillets, I.; Hamou, M.F.; Ruegg, C.; Stupp, R.; Delorenzi, M.; Hegi, M.E. Modulation of angiogenic and inflammatory response in glioblastoma by hypoxia. *PLoS One* **2009**, *4*, e5947, doi:10.1371/journal.pone.0005947.

50. Kickingereder, P.; Sahm, F.; Radbruch, A.; Wick, W.; Heiland, S.; Deimling, A.; Bendszus, M.; Wiestler, B. IDH mutation status is associated with a distinct hypoxia/angiogenesis transcriptome signature which is non-invasively predictable with rCBV imaging in human glioma. *Sci Rep* **2015**, *5*, 16238, doi:10.1038/srep16238.

51. Chen, W.; Cheng, X.; Wang, X.; Wang, J.; Wen, X.; Xie, C.; Liao, C. Clinical implications of hypoxia-inducible factor-1alpha and caveolin-1 overexpression in isocitrate dehydrogenase-wild type glioblastoma multiforme. *Oncol Lett* **2019**, *17*, 2867-2873, doi:10.3892/ol.2019.9929.

52. Metellus, P.; Colin, C.; Taieb, D.; Guedj, E.; Nanni-Metellus, I.; de Paula, A.M.; Colavolpe, C.; Fuentes, S.; Dufour, H.; Barrie, M., et al. IDH mutation status impact on in vivo hypoxia biomarkers expression: new insights from a clinical, nuclear imaging and immunohistochemical study in 33 glioma patients. *J Neurooncol* **2011**, *105*, 591-600, doi:10.1007/s11060-011-0625-2.

53. Lluis, J.M.; Buricchi, F.; Chiarugi, P.; Morales, A.; Fernandez-Checa, J.C. Dual role of mitochondrial reactive oxygen species in hypoxia signaling: activation of nuclear factor-{kappa}B via c-SRC and oxidant-dependent cell death. *Cancer Res* **2007**, *67*, 7368-7377, doi:10.1158/0008-5472.CAN-07-0515.

54. Kasznicki, J.; Sliwinska, A.; Drzewoski, J. Metformin in cancer prevention and therapy. *Ann Transl Med* **2014**, *2*, 57, doi:10.3978/j.issn.2305-5839.2014.06.01.

55. Seliger, C.; Luber, C.; Gerken, M.; Schaertl, J.; Proescholdt, M.; Riemenschneider, M.J.; Meier, C.R.; Bogdahn, U.; Leitzmann, M.F.; Klinkhammer-Schalke, M., et al. Use of metformin and survival of patients with high-grade glioma. *Int J Cancer* **2019**, *144*, 273-280, doi:10.1002/ijc.31783.

56. Seliger, C.; Genbrugge, E.; Gorlia, T.; Chinot, O.; Stupp, R.; Nabors, B.; Weller, M.; Hau, P.; Group, E.B.T. Use of metformin and outcome of patients with newly diagnosed glioblastoma: Pooled analysis. *Int J Cancer* **2019**, 10.1002/ijc.32337, doi:10.1002/ijc.32337.

57. Valtorta, S.; Lo Dico, A.; Raccagni, I.; Gaglio, D.; Belloli, S.; Politi, L.S.; Martelli, C.; Diceglie, C.; Bonanomi, M.; Ercoli, G., et al. Metformin and temozolomide, a synergic option to overcome resistance in glioblastoma multiforme models. *Oncotarget* **2017**, *8*, 113090-113104, doi:10.18632/oncotarget.23028.

58. Lee, J.E.; Lim, J.H.; Hong, Y.K.; Yang, S.H. High-Dose Metformin Plus Temozolomide Shows Increased Anti-tumor Effects in Glioblastoma In Vitro and In Vivo Compared with Monotherapy. *Cancer Res Treat* **2018**, *50*, 1331-1342, doi:10.4143/crt.2017.466.

59. Gao, L.B.; Tian, S.; Gao, H.H.; Xu, Y.Y. Metformin inhibits glioma cell U251 invasion by downregulation of fibulin-3. *Neuroreport* **2013**, *24*, 504-508, doi:10.1097/WNR.0b013e32836277fb.

60. Al Hassan, M.; Fakhoury, I.; El Masri, Z.; Ghazale, N.; Dennaoui, R.; El Atat, O.; Kanaan, A.; El-Sibai, M. Metformin Treatment Inhibits Motility and Invasion of Glioblastoma Cancer Cells. *Anal Cell Pathol (Amst)* **2018**, *2018*, 5917470, doi:10.1155/2018/5917470.

61. Miller, R.A.; Chu, Q.; Xie, J.; Foretz, M.; Viollet, B.; Birnbaum, M.J. Biguanides suppress hepatic glucagon signalling by decreasing production of cyclic AMP. *Nature* **2013**, *494*, 256-260, doi:10.1038/nature11808.

62. Andrzejewski, S.; Siegel, P.M.; St-Pierre, J. Metabolic Profiles Associated With Metformin Efficacy in Cancer. *Front Endocrinol (Lausanne)* **2018**, *9*, 372, doi:10.3389/fendo.2018.00372.

63. Guimaraes, T.A.; Farias, L.C.; Santos, E.S.; de Carvalho Fraga, C.A.; Orsini, L.A.; de Freitas Teles, L.; Feltenberger, J.D.; de Jesus, S.F.; de Souza, M.G.; Santos, S.H., et al. Metformin increases PDH and suppresses HIF-1alpha under hypoxic conditions and induces cell death in oral squamous cell carcinoma. *Oncotarget* **2016**, *7*, 55057-55068, doi:10.18632/oncotarget.10842.

64. Zhou, X.; Chen, J.; Yi, G.; Deng, M.; Liu, H.; Liang, M.; Shi, B.; Fu, X.; Chen, Y.; Chen, L., et al. Metformin suppresses hypoxia-induced stabilization of HIF-1alpha through reprogramming of oxygen metabolism in hepatocellular carcinoma. *Oncotarget* **2016**, *7*, 873-884, doi:10.18632/oncotarget.6418.

65. Zannella, V.E.; Dal Pra, A.; Muaddi, H.; McKee, T.D.; Stapleton, S.; Sykes, J.; Glicksman, R.; Chaib, S.; Zamiara, P.; Milosevic, M., et al. Reprogramming metabolism with metformin improves tumor oxygenation and radiotherapy response. *Clin Cancer Res* **2013**, *19*, 6741-6750, doi:10.1158/1078-0432.CCR-13-1787.

66. Lalau, J.D.; Race, J.M. Lactic acidosis in metformin therapy: searching for a link with metformin in reports of 'metformin-associated lactic acidosis'. *Diabetes Obes Metab* **2001**, *3*, 195-201.

67. Gerthofer, V.; Kreutz, M.; Renner, K.; Jachnik, B.; Dettmer, K.; Oefner, P.; Riemenschneider, M.J.; Proescholdt, M.; Vollmann-Zwerenz, A.; Hau, P., et al. Combined Modulation of Tumor Metabolism by Metformin and Diclofenac in Glioma. *Int J Mol Sci* **2018**, *19*, doi:10.3390/ijms19092586.

68. Griss, T.; Vincent, E.E.; Egnatchik, R.; Chen, J.; Ma, E.H.; Faubert, B.; Viollet, B.; DeBerardinis, R.J.; Jones, R.G. Metformin Antagonizes Cancer Cell Proliferation by Suppressing Mitochondrial-Dependent Biosynthesis. *PLoS biology* **2015**, *13*, e1002309, doi:10.1371/journal.pbio.1002309.

69. Kheirandish, M.; Mahboobi, H.; Yazdanparast, M.; Kamal, W.; Kamal, M.A. Anti-cancer Effects of Metformin: Recent Evidences for its Role in Prevention and Treatment of Cancer. *Curr Drug Metab* **2018**, *19*, 793-797, doi:10.2174/1389200219666180416161846.

70. Rotermund, C.; Machetanz, G.; Fitzgerald, J.C. The Therapeutic Potential of Metformin in Neurodegenerative Diseases. *Front Endocrinol (Lausanne)* **2018**, *9*, 400, doi:10.3389/fendo.2018.00400.

71. Liu, J.; Zhang, C.; Hu, W.; Feng, Z. Tumor suppressor p53 and its mutants in cancer metabolism. *Cancer Lett* **2015**, *356*, 197-203, doi:10.1016/j.canlet.2013.12.025.

72. Molina, J.R.; Sun, Y.; Protopopova, M.; Gera, S.; Bandi, M.; Bristow, C.; McAfoos, T.; Morlacchi, P.; Ackroyd, J.; Agip, A.A., et al. An inhibitor of oxidative phosphorylation exploits cancer vulnerability. *Nat Med* **2018**, *24*, 1036-1046, doi:10.1038/s41591-018-0052-4.

73. Shi, Y.; Lim, S.K.; Liang, Q.; Iyer, S.V.; Wang, H.Y.; Wang, Z.; Xie, X.; Sun, D.; Chen, Y.J.; Tabar, V., et al. Gboxin is an oxidative phosphorylation inhibitor that targets glioblastoma. *Nature* **2019**, *567*, 341-346, doi:10.1038/s41586-019-0993-x.

74. Ben Sahra, I.; Regazzetti, C.; Robert, G.; Laurent, K.; Le Marchand-Brustel, Y.; Auberger, P.; Tanti, J.F.; Giorgetti-Peraldi, S.; Bost, F. Metformin, independent of AMPK, induces mTOR inhibition and cell-cycle arrest through REDD1. *Cancer Res* **2011**, *71*, 4366-4372, doi:10.1158/0008-5472.CAN-10-1769.

75. Dallaglio, K.; Bruno, A.; Cantelmo, A.R.; Esposito, A.I.; Ruggiero, L.; Orecchioni, S.; Calleri, A.; Bertolini, F.; Pfeffer, U.; Noonan, D.M., et al. Paradoxic effects of metformin on endothelial cells and angiogenesis. *Carcinogenesis* **2014**, *35*, 1055-1066, doi:10.1093/carcin/bgu001.

| 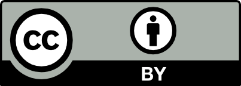 | © 2020 by the authors. Submitted for possible open access publication under the terms and conditions of the Creative Commons Attribution (CC BY) license (http://creativecommons.org/licenses/by/4.0/). |
| --- | --- |
