# Supplementary material for: An Experimentally Defined Hypoxia Gene Signature in Glioblastoma and Its Modulation by Metformin
Source: Biology (Basel). 2020 Sep 2;9(9):264. doi: 10.3390/biology9090264 (PMC7563149; doi:10.3390/biology9090264)
Supplement: Supplementary file 1 [file biology-09-00264-s001.zip › Calvo-Tardon_Additional file 1-supplementary legends.docx]

**Additional file 1. Supplementary Figure and Table Legends**

**Figure S1**

Whole transcriptome analysis from human GBM cell lines exposed to different oxygen conditions. **a** Representative example of western blot of nuclear stabilization of HIF-1α and loading control TATA-binding protein (TBP) of human GBM cell line Ge835 at 21%, 5%, and 1% O_2_. **b** Venn diagram of unique genes modulated under hypoxia compared to physioxia for each individual cell line (p<0.05, FC≥1.39). **c** Unsupervised clustering of all samples (five cell lines exposed to three oxygen conditions; mean of three biological replicates). **d** Affymetrix microarray probe and gene ID of the 36 genes of the hypoxia gene signature. **e** List of reported HRE-containing genes from our hypoxia signature

**Figure S2**

Functional assays of human GBM cell lines and astrocytes exposed to metformin. **a** Effect of 10mM metformin on viable cell number under 21% and 1% oxygen; **b** Effect of increasing concentrations of metformin on viable cell number of astrocytes under 21%, 5% and 1% oxygen (mean of 3 independent experiments +/- SD; 2-way ANOVA, Sidak’s adjusted p-value. **p<0.01, ****p<0.0001).

**Figure S3**

Effect of differential oxygen availability and metformin on oxygen consumption rate (OCR) and extracellular acidification rate (ECAR) on human and mouse GBM cell lines. **a** Detailed Seahorse XF Cell Mito Stress test profiles on OCR data of Ge835 GBM cell line at 21%, 5%, and 1% O_2_ following exposure to vehicle or different concentrations of metformin. **b** Basal OCR and (**c**) basal ECAR of all cell lines tested (n=3; mean +/- SD; 2-way ANOVA).

**Figure S4**

Western blot image and densitometry data for HIF-1α expression by GBM line Ge835 under different oxygen fractions. Raw image data used for Figure S1a.Western blot image of the transfer membrane showing the total nuclear fraction of HIF1α and TBP (nuclear localization reference) protein levels for Ge835 cells incubated at 21%, 5% and 1% O2. Band intensities were calculated as a ratio of HIF1α to TBP and analyzed using ImageJ software 1.52 (Open Software).

**Table S1**

Relative gene expression values used for heat map data in Figure S1c. Gene list and respective fold change values from 3 independent experiments with a t-test for each cell line. Median value is reported for genes with multiple probes.

**Table S2**

Fold change values of metformin modulation of the hypoxia gene signature used for heat map in Fig 5, p-values of the comparison hypoxia versus physioxia and relative standard deviations.

**Table S3.**

Univariate and multivariate analysis of the hypoxia signature.
