# Supplementary material for: An Experimentally Defined Hypoxia Gene Signature in Glioblastoma and Its Modulation by Metformin
Source: Biology (Basel). 2020 Sep 2;9(9):264. doi: 10.3390/biology9090264 (PMC7563149; doi:10.3390/biology9090264)
Supplement: Supplementary file 1 [file biology-09-00264-s001.zip › Table S3.pdf]

Table S3. Univariate and multivariate analysis of the hypoxia signature.

	Univariate		Multivariate	
**Variable**	**HR**	**p-value**	**HR**	**p-value**
**MGMT gene promoter**<br>methylated vs unmethylated	0.686	0.002	0.772	0.053
**IDH gene status**<br>Mutant vs wt	0.344	<0.001	0.362	<0.001
**Hypoxia signature**<br>High vs low expression	1.397	0.002	1.227	0.170
