# Supplementary figures and images for: An Experimentally Defined Hypoxia Gene Signature in Glioblastoma and Its Modulation by Metformin
Source: Biology (Basel). 2020 Sep 2;9(9):264. doi: 10.3390/biology9090264 (PMC7563149; doi:10.3390/biology9090264)

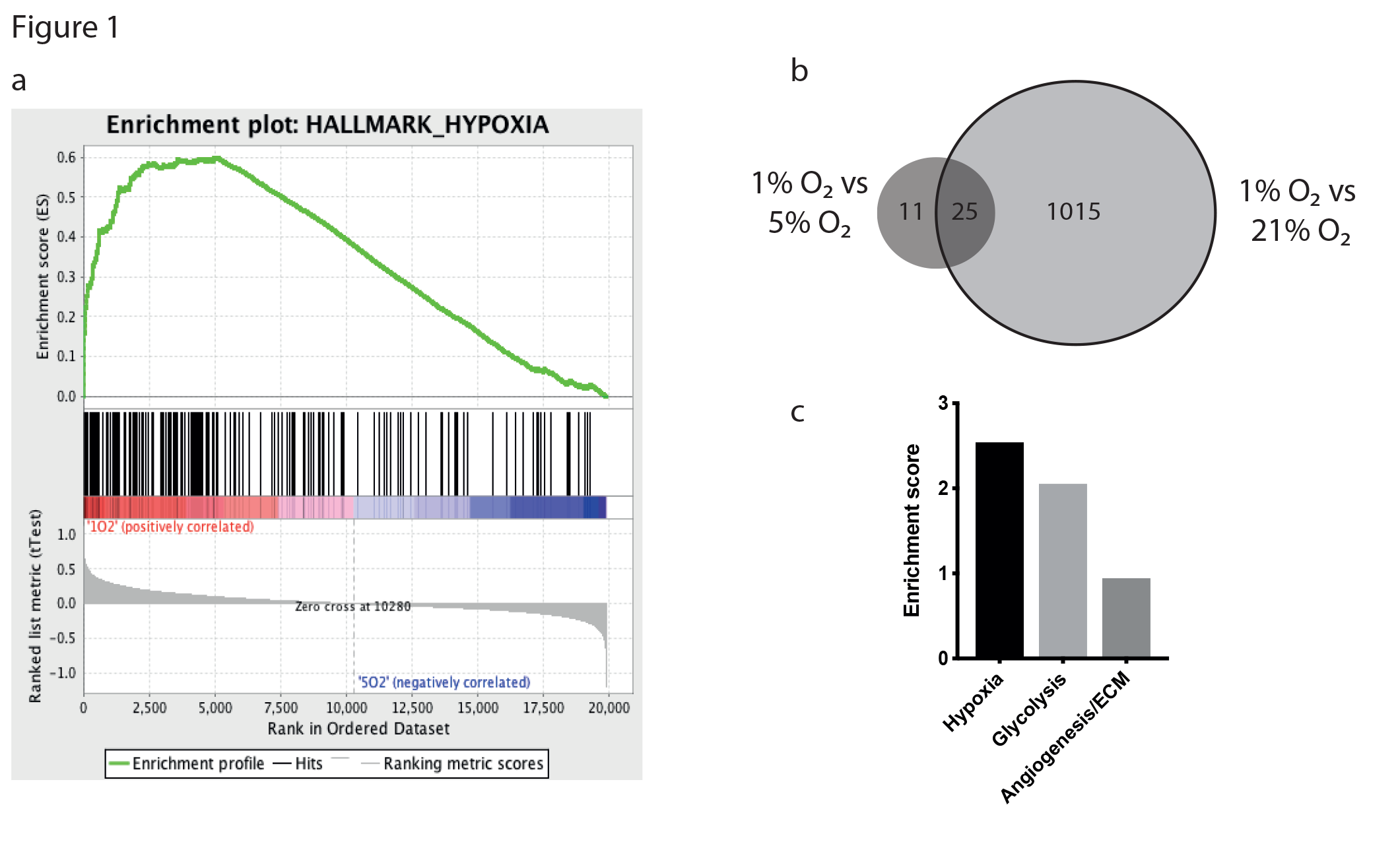

Supplement: Supplementary file 1 [file biology-09-00264-s001.zip › Calvo Tardon et al. Fig1.tif]

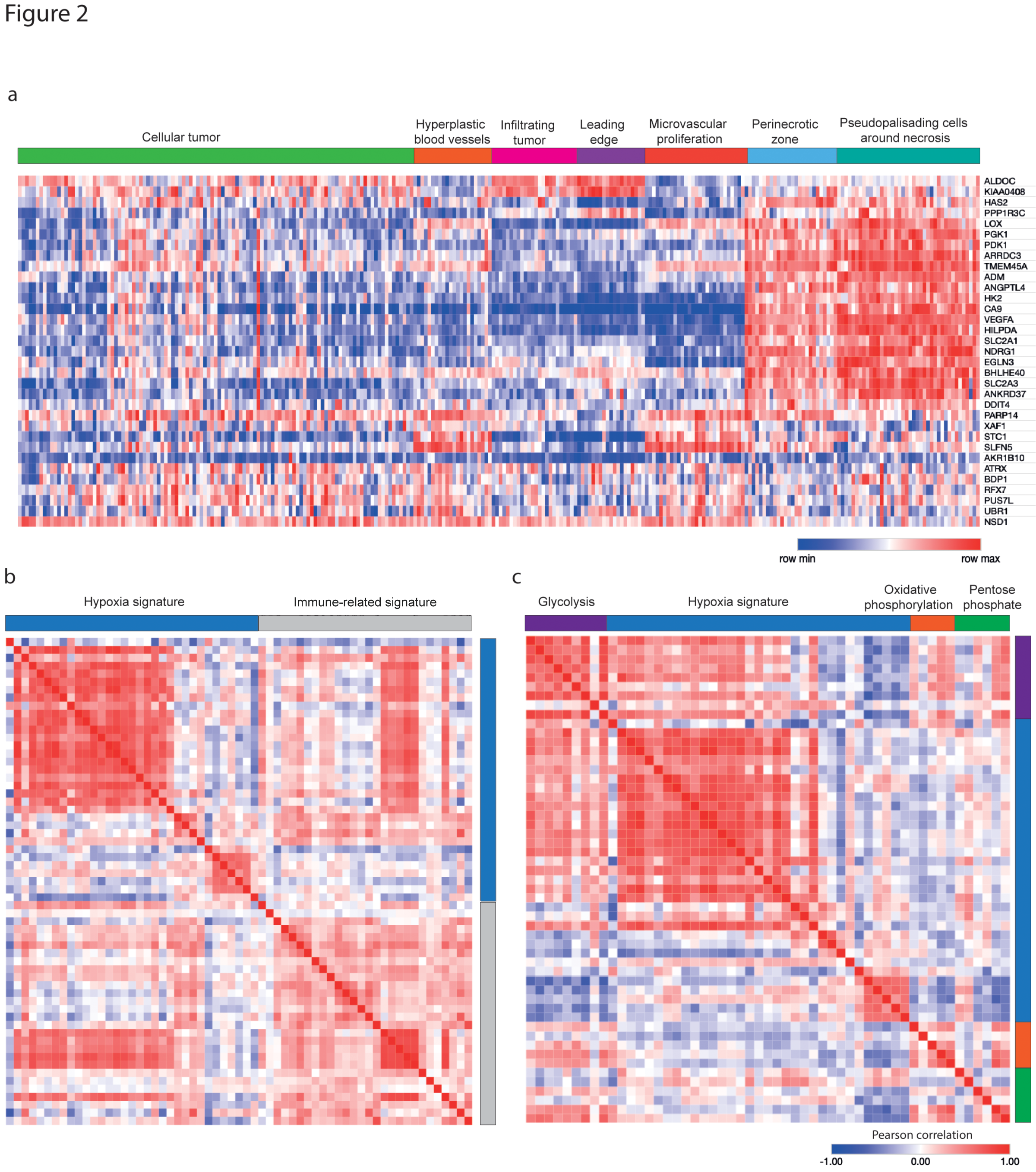

Supplement: Supplementary file 1 [file biology-09-00264-s001.zip › Calvo Tardon et al. Fig2.tif]

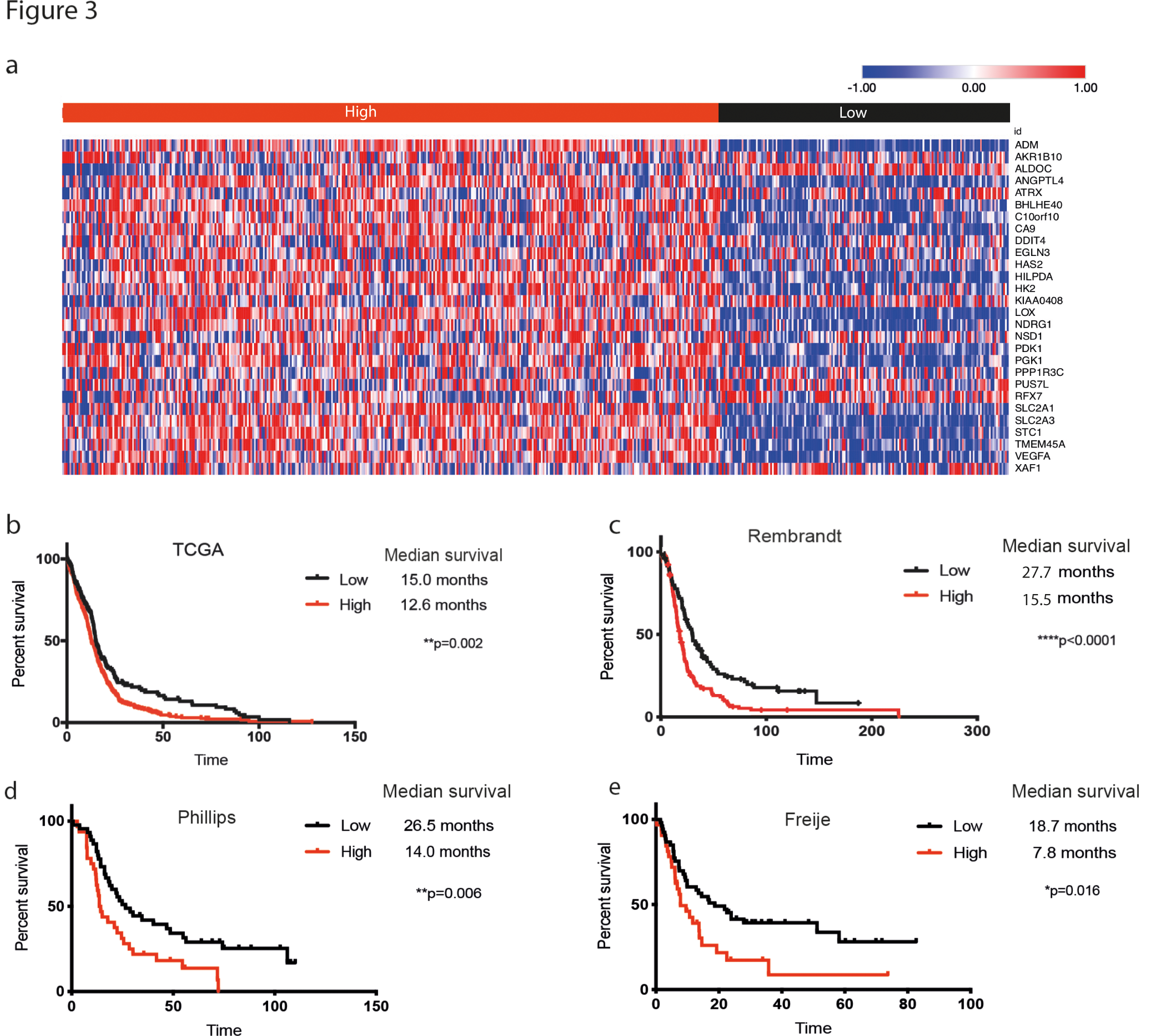

Supplement: Supplementary file 1 [file biology-09-00264-s001.zip › Calvo Tardon et al. Fig3.tif]

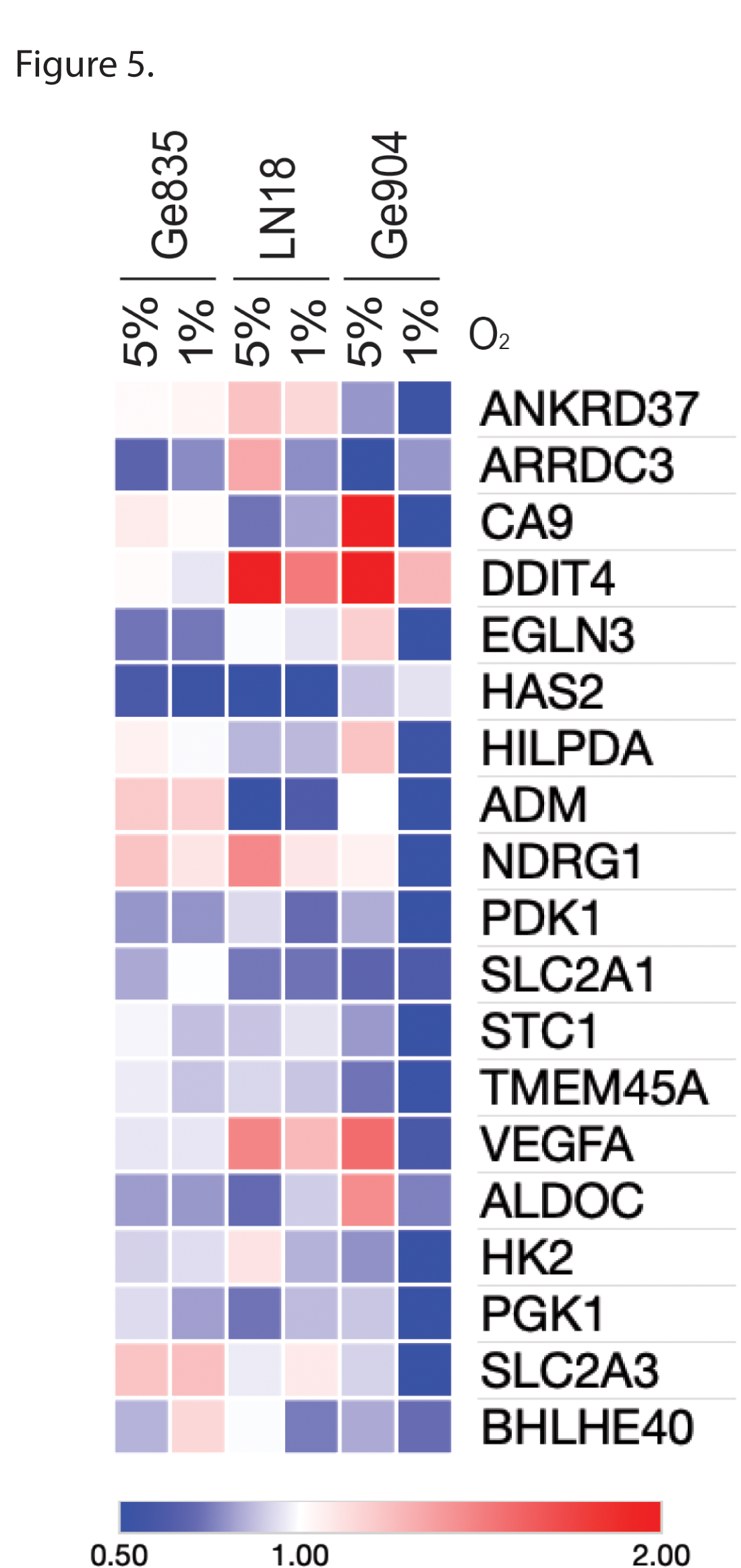

Supplement: Supplementary file 1 [file biology-09-00264-s001.zip › Calvo Tardon et al. Fig5.tif]

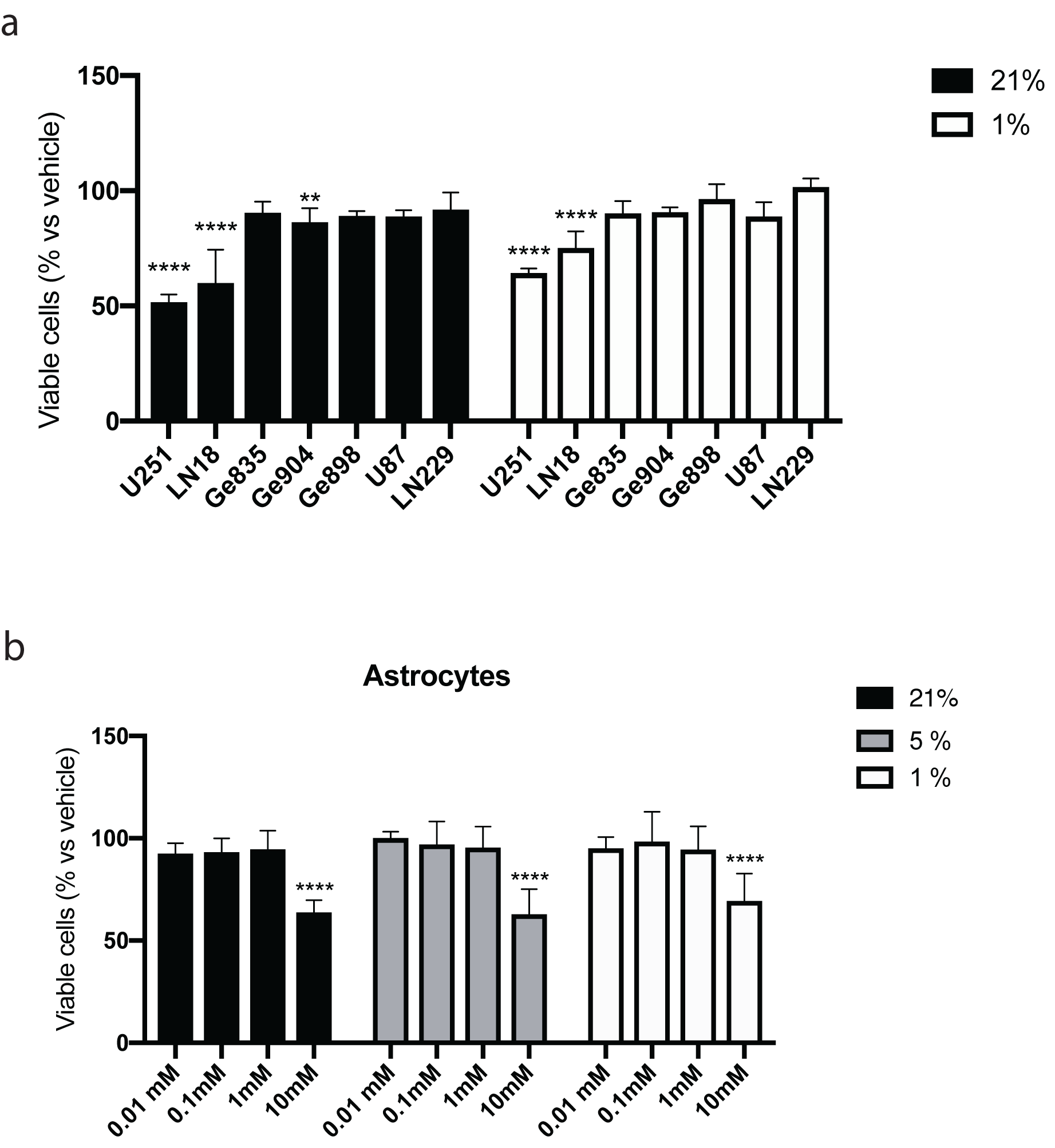

Supplement: Supplementary file 1 [file biology-09-00264-s001.zip › Calvo Tardon et al. FigS2.tif]

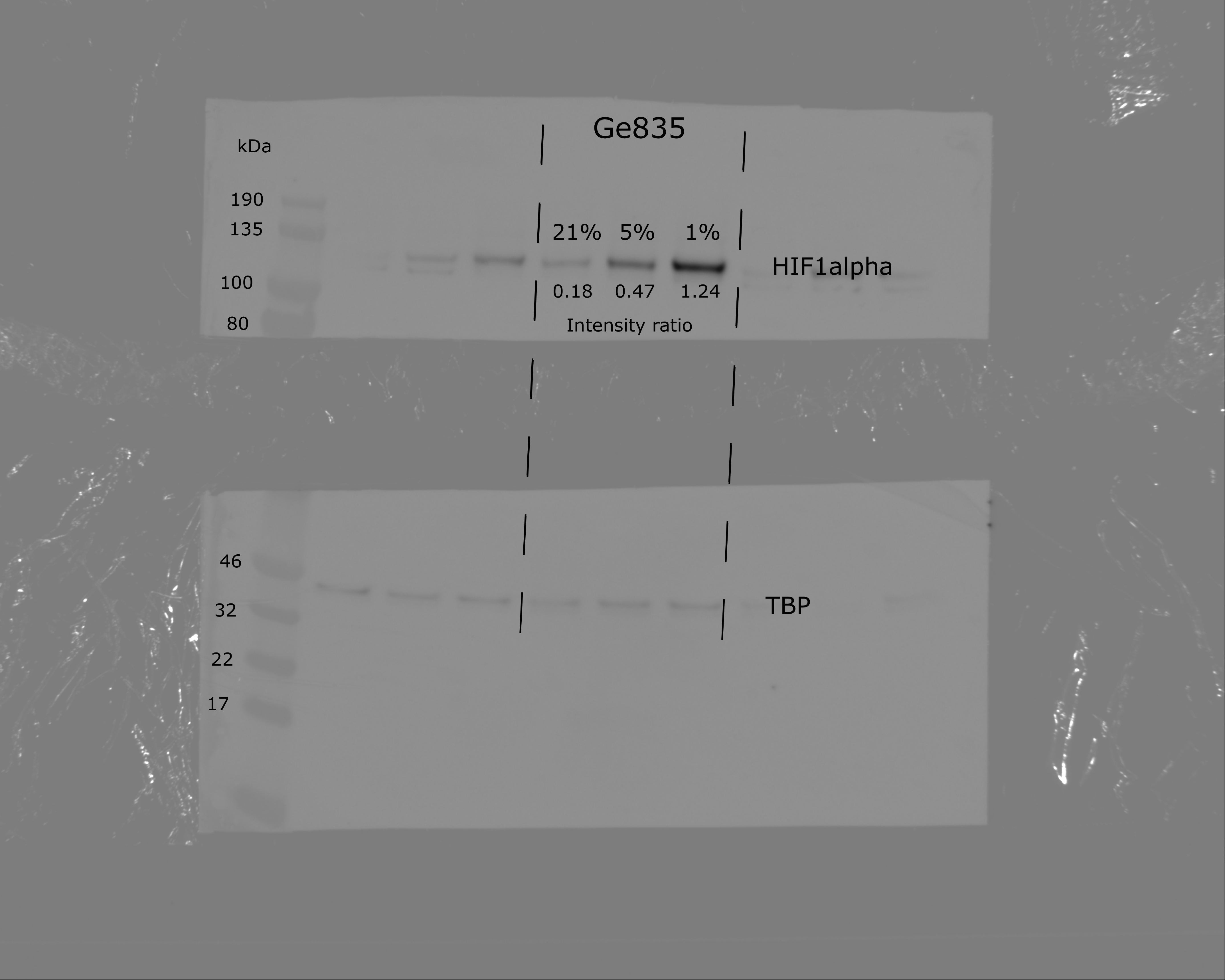

Supplement: Supplementary file 1 [file biology-09-00264-s001.zip › Calvo Tardon et al. FigS4.tif]
